# Host–parasite cross-species regulation by *Schistosoma japonicum* EV-miRNA sja-miR-2a-3p limits hepatic fibrosis via BCL2 suppression in hepatic stellate cells

**DOI:** 10.1186/s13567-026-01794-y

**Published:** 2026-08-14

**Authors:** Bowen Dong, Danlin Zhu, Junhan Xiong, Yuanzhao Sun, Ruiting Zhang, Zhiqiang Fu, Jinming Liu, Haoran Zhong, Yamei Jin

**Affiliations:** 1 National Reference Laboratory for Animal Schistosomiasis, Key Laboratory of Animal Parasitology of Ministry of Agriculture and Rural Affairs, Shanghai Veterinary Research Institute, Chinese Academy of Agricultural Sciences, Shanghai, China; 2 College of Animal Science and Veterinary Medicine, Shanxi Agricultural University, Shanxi, China

**Keywords:** *Schistosoma japonicum*, extracellular vesicles, MiRNA, liver fibrosis, hepatic stellate cell, apoptosis

## Abstract

Schistosomiasis remains an important zoonotic parasitic disease, and hepatic fibrosis is a major cause of chronic pathology following *Schistosoma japonicum* infection. In schistosomiasis, hepatic fibrosis is the major cause of severe pathological damage. However, during the long-term co-evolution and coexistence between *Schistosoma* parasites and their hosts, the parasite can modulate the host hepatic fibrotic process through extracellular vesicle (EV)-mediated cross-species communication. In this study, we identified an EV-derived microRNA from *Schistosoma japonicum*, sja-miR-2a-3p, that suppresses hepatic pathology in the host. Using transwell co-culture and EV-isolation experiments, we confirmed that the helminth-specific sja-miR-2a-3p could be transferred to hepatic stellate cells (HSCs) via EVs. Bioinformatic and dual-luciferase assays revealed that host BCL2, a major anti-apoptotic gene, is a direct target of sja-miR-2a-3p. In vitro, sja-miR-2a-3p overexpression suppressed HSC proliferation and migration and induced mitochondrial dysfunction, caspase-3/-7/-9 activation, and apoptosis. In vivo, administration of sja-miR-2a-3p agomir to *S. japonicum*-infected mice significantly downregulated hepatic BCL2 expression, enhanced HSC apoptosis, alleviated granuloma formation, collagen deposition, and inflammation. Collectively, our findings reveal that schistosome EV-delivered sja-miR-2a-3p attenuates liver fibrosis by promoting mitochondrial apoptosis and functionally suppressing HSC activation. This study supports sja-miR-2a-3p as a candidate antifibrotic molecule.

**Graphical Abstract:**

**Figure:**
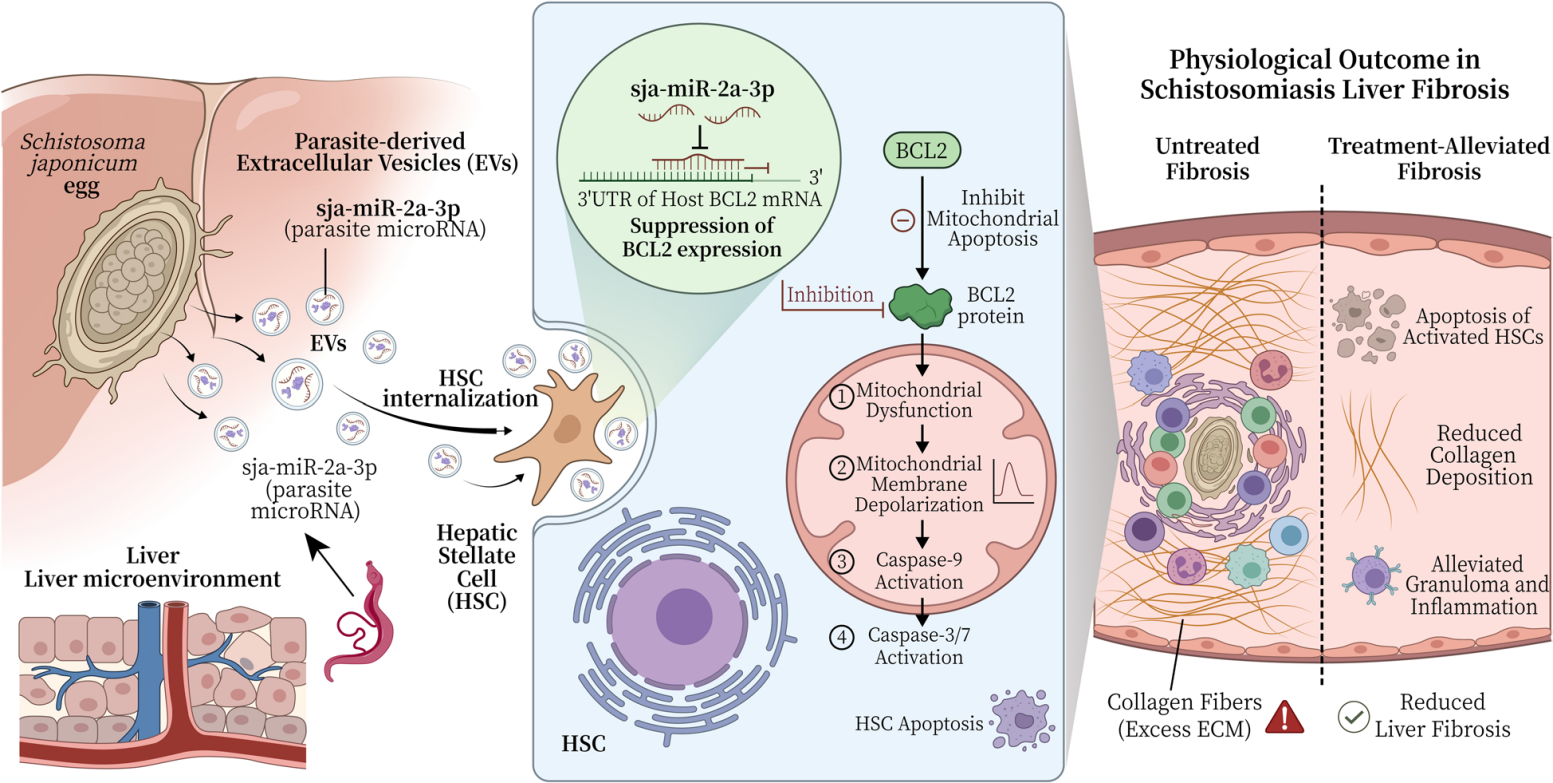

**Supplementary Information:**

The online version contains supplementary material available at 10.1186/s13567-026-01794-y.

## Introduction

Schistosomiasis is a zoonotic parasitic disease of major importance to both public health and veterinary medicine [1, 2]. Among the causative species, *Schistosoma japonicum* is unique because of its broad host range, infecting numerous mammalian reservoir hosts including livestock, rodents, and humans [3–5]. These animal reservoirs play a critical role in sustaining parasite transmission and maintaining endemicity in many regions of tropical and subtropical regions [6]. As a consequence, schistosomiasis remains a persistent health challenge, with hundreds of millions of people at risk of infection worldwide. Chronic infection with *S. japonicum* is characterized by severe hepatointestinal pathology, of which hepatic fibrosis represents one of the most prominent and clinically significant outcomes [7]. The development of hepatic fibrosis is primarily driven by host immune responses to parasite eggs deposited in the liver, which trigger sustained inflammatory reactions and tissue remodeling [8]. A central cellular event in this process is the activation of hepatic stellate cells (HSCs), which undergo transdifferentiation into myofibroblast-like cells and produce large amounts of extracellular matrix (ECM) proteins, including type I and type III collagens and α-smooth muscle actin (α-SMA). Progressive ECM accumulation disrupts normal liver architecture and contributes to chronic hepatic pathology associated with schistosomiasis [9].

During the long-term co-existence of helminth and their hosts, extracellular vesicles (EVs) released by helminth have emerged as important mediators of cross-species communication [10]. These nanoscale vesicles are capable of delivering diverse biomolecules—including proteins, lipids, and regulatory RNAs—to host cells, thereby modulating immune responses and disease progression [11, 12]. Accumulating evidence indicates that schistosome egg-derived EVs can release multiple microRNAs (miRNA), including sja-miR-1 [13], sja-miR-71a [14], sja-miR-2162 [15], novel miRNA-30 [16], and miRNA-33 [17]; these miRNAs have been shown to influence hepatic fibrosis, in part by regulating the TGF-β/Smad signaling cascade. Nevertheless, the functional contributions of many other bioactive molecules within schistosome-derived EVs remain largely unexplored, raising the possibility that additional schistosome miRNAs may shape HSC behavior and thereby impact fibrogenesis or its resolution [18].

Supporting this possibility, previous studies have demonstrated that eight schistosome-derived miRNAs, including sja-miR-2a-3p, can be detected in primary HSCs isolated from mice infected with *S. japonicum* [15]. Consistently, in our earlier work using a transwell co-culture system of schistosomes and HSCs, small RNA sequencing revealed abundant reads of sja-miR-2a-3p, suggesting that schistosomes may deliver this miRNA to HSCs through *Sj*EVs [19]. These observations imply that sja-miR-2a-3p may be functionally involved in modulating HSC activity during infection, although its mechanistic role has not been fully clarified.

In the present study, we demonstrate that sja-miR-2a-3p released from *Sj*EVs is transferred into host HSCs and directly targets the anti-apoptotic gene BCL2, thereby activating mitochondrial apoptosis and reducing liver fibrosis. These findings provide new insight into parasite–host molecular communication and identify a helminth-derived miRNA as a potential antifibrotic therapeutic candidate.

## Materials and methods

### Ethics statement

All animal experiments were conducted in accordance with institutional guidelines and were approved by the Ethics and Animal Welfare Committee of the Shanghai Veterinary Research Institute, Chinese Academy of Agricultural Sciences (permit no. SYXK-20160010; approval no. SV-20241011–01).

### Animals, parasites, and infection

Six-week-old male SPF BALB/c mice were purchased from Shanghai Jiesijie Laboratory Animal Co., Ltd. (Shanghai, China) and housed under standard conditions with free access to food and water. Mice were randomly assigned to experimental groups. The *S. japonicum* Anhui strain was maintained at the Shanghai Veterinary Research Institute. Mice were percutaneously infected with a defined number of cercariae applied to the shaved abdominal skin [20]. At designated time points, mice were sacrificed and adult worms were recovered by hepatic perfusion as previously described [20, 21].

### Cell culture and transfection

Human hepatic stellate cells (LX-2) and HEK293T cells were obtained from Boster Biological Technology (Wuhan, China). LX-2 cells were cultured in high-glucose Dulbecco’s modified Eagle medium (DMEM) supplemented with 15% fetal bovine serum (FBS) and 1% penicillin–streptomycin, while HEK293T cells were maintained in DMEM containing 10% FBS. Cells were incubated at 37 °C with 5% CO₂.

For transfection, LX-2 cells were seeded in 12-well plates (5 × 10^5^ cells/well) and transfected with 40 pmol of sja-miR-2a-3p mimics, inhibitors, or corresponding negative controls (GenePharma, China) using Lipofectamine 3000 (Invitrogen, USA). A mock group received transfection reagent alone. miRNA mimcs sequences are listed in Additional file 1. For EV stimulation, transfected cells were treated with *Sj*EVs (2.96 × 10⁹ particles/mL) for 6 h. Where indicated, cells were pretreated with dynasore (150 μM) for 30 min before incubation with *Sj*EVs or *Sj*EV-depleted products. Cells were harvested 48 h post-transfection for downstream analyses.

### Establishment of a transwell model for LX-2 cells

*S. japonicum* eggs were isolated as previously described [22, 23]. Transwell co-culture systems were established in 12-well plates using 0.4-μm pore-size polyethylene terephthalate (PET) inserts (Corning, USA). LX-2 cells (5 × 10^5^ cells/well) were seeded in the lower chamber with DMEM containing 10% FBS and 1% penicillin–streptomycin. In the upper chamber, 10^4^ *S. japonicum* eggs were added in fresh medium, while control wells received egg-processing medium alone. After 48 h of co-culture, LX-2 cells were harvested for quantitative polymerase chain reaction (qPCR) analysis.

### Isolation and purification of *Sj*EVs

Six-week-old male BALB/c mice were infected with ~100 *S. japonicum* cercariae. At 30 days post-infection, adult worms were recovered by hepatic perfusion and cultured in Roswell Park Memorial Institute (RPMI)−1640 at 37 °C with 5% CO₂. Worm culture supernatants were collected every 2 h, centrifuged at 3000 *g* for 10 min, and filtered through a 0.22-μm membrane to remove debris.

Supernatants were concentrated using the Exosupur^®^ High Concentration Kit (Echo Biotech, China) and further purified by size-exclusion chromatography with the Exosupur^®^ Exosome Purification Kit according to the manufacturer’s instructions. Early fractions were collected as EV-depleted excretory/secretory products (ESPs), whereas EV-enriched fractions were pooled and concentrated using a 100-kDa centrifugal filter. Purified *Sj*EVs were stored at −80 °C until use. Detailed procedures are provided in Additional file 2 in accordance with EV study guidelines [24].

### *Sj*EV uptake assay

Isolated *Sj*EVs were labeled with PKH67 (Sigma Aldrich, USA) according to the manufacturer’s instructions. Briefly, EVs were stained in Diluent C for 5 min at room temperature, and the reaction was terminated with 1% bovine serum albumin (BSA). Labeled EVs were re-purified by ultracentrifugation at 100 000 *g* for 90 min and washed with PBS to remove excess dye. PKH67-stained *Sj*EV-depleted ESPs were used as labeling controls. PKH67-labeled *Sj*EVs were incubated with LX-2 cells for 2 h at 37 °C. Where indicated, cells were pretreated with dynasore (150 μM) for 30 min prior to EV incubation. After washing with PBS, cells were fixed with 4% paraformaldehyde, stained with TRITC-phalloidin and 4′,6-diamidino-2-phenylindole (DAPI), and visualized by fluorescence microscopy (Olympus, Japan).

### Electron microscopy and nanoparticle tracking analyses (NTA)

*Sj*EVs and *Sj*EV-depleted ESPs were adsorbed onto formvar-coated grids, negatively stained with 2% phosphotungstic acid, and visualized using a transmission electron microscope (FEI, the Netherlands). Particle size distribution and concentration were analyzed by NTA using the ZetaView system (Particle Metrix, Germany).

### Protein extraction, peptide digestion, and LC–MS/MS data acquisition

Purified *Sj*EVs were lysed in SDT buffer, and proteins were digested using the Filter-Aided Sample Preparation (FASP) method as previously described [18]. Peptides were analyzed by liquid chromatography–tandem mass spectrometry (LC–MS/MS), and data were processed using MaxQuant (v1.5.5.1) against the *Schistosoma japonicum* UniProtKB database (downloaded 6 March 2025). Protein identification required at least two unique peptides with a false discovery rate (FDR) < 0.01.

### Bioinformatics analysis of miRNA targets

Two bioinformatic analysis software, miRanda [25] and RNAhybrid [26] were applied to predict mRNA targets of sja-miR-2a-3p in *Homo sapiens*. The mRNAs were based off the latest dataset of *H.sapiens* whole transcriptome (GenBank accession no. PRJNA1451788). The miRNA potential targeted genes predicted by both software were selected for the following Gene Ontology (GO) [27] analysis via Metascape [28]. Afterward, BCL2 (GenBank accession no. NM_000657) was selected for further verification.

### Dual-luciferase reporter assay

Wild-type or mutant BCL2 3′UTR sequences were cloned into the pmirGLO vector (Promega, USA). HEK293T cells were co-transfected with sja-miR-2a-3p mimics or negative control (NC) and the corresponding reporter plasmids using Lipofectamine 3000. Luciferase activity was measured 48 h post-transfection using a Dual-Luciferase Reporter Assay Kit (Promega, USA) according to the manufacturer’s instructions.

### Flow cytometry assay

Apoptosis of LX-2 cells was assessed 48 h after transfection using an Annexin V/PI apoptosis detection kit (Yeasen, China). Cells were stained according to the manufacturer’s protocol and analyzed by flow cytometry. The percentages of early and late apoptotic cells were quantified.

### TUNEL staining

Apoptosis of LX-2 cells transfected with sja-miR-2a-3p mimics was evaluated using a TUNEL assay kit (Yeasen, China) according to the manufacturer’s instructions. Cells were fixed, permeabilized, and incubated with TdT reaction mixture. Nuclei were counterstained with DAPI, and TUNEL-positive cells were visualized by fluorescence microscopy.

### Mitochondrial morphology and membrane potential assessment

To evaluate mitochondrial alterations, LX-2 cells transfected with sja-miR-2a-3p mimics or NC were analyzed by transmission electron microscopy (TEM) and fluorescence staining. For ultrastructural analysis, cells were fixed in glutaraldehyde, embedded, and examined under a TEM. Mitochondrial morphology was assessed using MitoTracker Red CMXRos staining, with carbonyl cyanide *m*-chlorophenyl hydrazone (CCCP) treatment as a positive control. Fluorescence images were acquired by confocal microscopy. Mitochondrial membrane potential was measured using JC-1 staining (Yeasen, China) according to the manufacturer’s protocol. CCCP-treated cells served as depolarization controls. JC-1 monomer (green) and aggregate (red) fluorescence signals were detected by fluorescence microscopy.

### Caspase-3 activity assay

The relative level of caspase-3 activity was determined according to the instruction manual for Caspase-3 Activity Assay Kit (Beyotime, China). At the same time, another 5-μL sample was taken to determine the protein concentration by Bradford method.

### Cell proliferation assay

LX-2 cells transfected with NC or sja-miR-2a-3p mimics were seeded into 96-well plates and cultured for 24, 48, 72, and 96 h. Cell proliferation was assessed using a CCK-8 assay (Dojindo, Japan) according to the manufacturer’s instructions. Absorbance at 450 nm was measured using a microplate reader (Bio-Tek, USA).

### EdU proliferation assay

Cell proliferation was assessed using the BeyoClick™ EdU Cell Proliferation Kit with Alexa Fluor 488 (C0071S, Beyotime, China) according to the manufacturer’s instructions. Fluorescence images were captured using a confocal microscope (ZEISS, Germany). Three random fields per sample were analyzed, and the percentage of EdU-positive cells was calculated.

### Wound healing assay

LX-2 cells (1 × 10⁶) were seeded in six-well plates and cultured overnight. Cells were then transfected with NC or sja-miR-2a-3p mimics as described above. After reaching approximately 90–100% confluence, a linear scratch was created using a sterile 200-μL pipette tip. Detached cells were removed by washing with PBS, and cells were maintained in DMEM containing 1% FBS. Images of the wound area were captured at 0 and 48 h using a light microscope. The migration rate was calculated on the basis of wound closure area using ImageJ software (version 1.52a, NIH, USA). The relative migration area was determined by comparing the wound closure in the experimental group with that of the NC group.

### Western blot

Cells were lysed in radioimmunoprecipitation assay (RIPA) buffer containing protease and phosphatase inhibitors. Protein concentrations were determined using a bicinchoninic acid (BCA) assay (Yeasen, China). Equal amounts of protein (20 μg) were separated by 10% sodium dodecyl sulfate (SDS)–polyacrylamide gel electrophoresis (PAGE) and transferred onto polyvinylidene fluoride (PVDF) membranes (Bio-Rad, USA). After blocking with 5% BSA, membranes were incubated overnight at 4 °C with primary antibodies against BCL2, cleaved caspase-3, caspase-7, and caspase-9 (Proteintech, China). After incubation with horseradish peroxidase (HRP)-conjugated secondary antibodies, signals were detected using enhanced chemiluminescence (ECL) reagents and visualized with a chemiluminescence imaging system (Bio-Rad, USA). β-Actin or β-tubulin (Servicebio, China) served as loading controls. Antibody details are listed in Additional file 3.

### RNA-sequencing and bioinformatics analysis of DEGs

Messenger RNA (mRNA) libraries were prepared using the Optimal Dual-mode mRNA Library Prep Kit (BGI-Shenzhen, China) and sequenced with three biological replicates per group (*n* = 9 libraries total). Raw reads were quality-filtered using SOAPnuke [29], and clean reads were aligned to the human reference genome (GRCh38) using HISAT2 [30]. Transcript assembly and mapping were performed with Bowtie2, and fusion genes and alternative splicing events were analyzed using Ericscript [31] and rMATS (V4.1.2) [32], respectively. Bowtie2 [33] was employed to map the clean reads to a comprehensive gene set, which included known and novel transcripts, as well as both coding and noncoding transcripts. Essentially, differential expression analysis was performed using the DESeq2 (v1.34.0) [34]. Genes with adjusted *Q*-values ≤ 0.05 and |log₂FoldChange|> 1 were considered significantly differentially expressed. Functional enrichment analyses of differentially expressed genes (DEGs) were performed using GO and Kyoto Encyclopedia of Genes and Genomes (KEGG) pathway analyses. Multiple testing correction was conducted using the Benjamini–Hochberg method to control the false discovery rate (FDR).

### Establishment of the *S. japonicum* liver fibrosis model in BALB/c mice

BALB/c mice were percutaneously infected with 100 ± 2 *S. japonicum* cercariae and randomly assigned to three groups (*Sj*, *Sj* + NC agomir, *Sj* + sja-miR-2a-3p agomir; *n* = 5 per group). At 4 weeks post-infection (wpi), mice were treated with praziquantel (PZQ; 500 mg/kg/day) by oral gavage for four consecutive days to eliminate adult worms and synchronize egg deposition. Beginning 2 days after PZQ treatment, mice received weekly tail vein injections of NC or sja-miR-2a-3p agomir for 4 weeks. The *Sj* group received equal volumes of PBS. Uninfected mice treated with PZQ and PBS served as controls. A total of 4 weeks after agomir administration, blood and major organs were collected for subsequent analyses.

### Liver, spleen index, and hematological analyses

Whole spleens and livers were collected, and their sizes were measured. The livers and spleens were weighed, and liver and spleen indices were calculated as the ratio of organ weight to body weight [35]. Blood samples (~150 μL/mouse) were collected into K2 EDTA tubes (Solarbio, China), and complete blood count (CBC) assay was conducted on a Mindray BC-6800 Plus analyzer (Mindray, China).

### Determination of hydroxyproline content

The extent of fibrosis in liver samples from each group was quantified using a hydroxyproline assay kit (Nanjing Jiancheng Bioengineering Institute, China) according to the manufacturer’s instructions.

### Histological, immunohistochemistry, and immunofluorescence analysis

Liver and major organs were fixed in 4% formaldehyde, paraffin-embedded, and sectioned (5 μm). Tissue morphology was assessed by hematoxylin and eosin (H&E) staining, and liver fibrosis was evaluated using Masson’s trichrome and Sirius Red staining (Servicebio, China). Fibrosis and inflammation were scored according to the Ishak index. Granuloma area and collagen-positive staining were quantified using ImageJ (NIH, USA). For immunohistochemistry, liver sections were incubated with primary antibodies against α-SMA, Col1α1, and Col3α1, followed by appropriate secondary antibodies. Positive staining areas were quantified using ImageJ. Immunofluorescence staining was performed to assess HSC apoptosis using antibodies against desmin and FasL. Nuclei were counterstained with DAPI, and images were captured using a fluorescence microscope (Olympus, Japan). Quantification was performed using ImageJ. Antibody details are listed in Additional file 3.

### FISH

Fluorescence in situ hybridization (FISH) was performed to examine the spatial relationship between sja-miR-2a-3p and BCL2 in liver sections. After antigen retrieval and proteinase K treatment, sections were prehybridized and incubated overnight with specific probes for sja-miR-2a-3p and BCL2 (500 nM) in a humidified chamber. Following stringent saline–sodium citrate (SSC) washes, nuclei were counterstained with DAPI. Fluorescence signals were visualized using a fluorescence microscope (Olympus, Japan). Probe sequences are listed in Additional file 4.

### RNA extraction and mRNA/miRNA quantification

Total RNA from LX-2 cells and liver tissues was extracted using TRNzol reagent (TIANGEN, China) and quantified by NanoDrop. For mRNA analysis, complementary DNA (cDNA) was synthesized using a reverse transcription kit (Yeasen, China) and subjected to SYBR Green-based qPCR on a LightCycler 96 system (Roche, China), with β-actin as the internal control.

For miRNA analysis, reverse transcription was performed using a stem-loop miRNA cDNA synthesis kit (Sangon Biotech, China), followed by SYBR Green qPCR with U6 as the reference gene. Serum miRNAs were isolated using a miRcute Serum/Plasma miRNA Isolation Kit (TIANGEN, China) and analyzed as described above.

Relative expression levels of mRNAs and miRNAs were calculated using the 2^−ΔΔCt^ method [36]. All reactions were performed in triplicate. Primer sequences are provided in Additional file 5.

### Statistical analysis

Data were analyzed with SPSS 25.0 software (SPSS Inc., USA) and expressed as mean ± standard deviation (SD) of three independent biological replicates. Data were statistically analyzed with Student’s *t*-tests. A *p*-value of < 0.05 was considered statistically significant in statistical analysis.

## Results

### sja-miR-2a-3p is delivered into HSCs via *Sj*EVs

**Figure 1:**
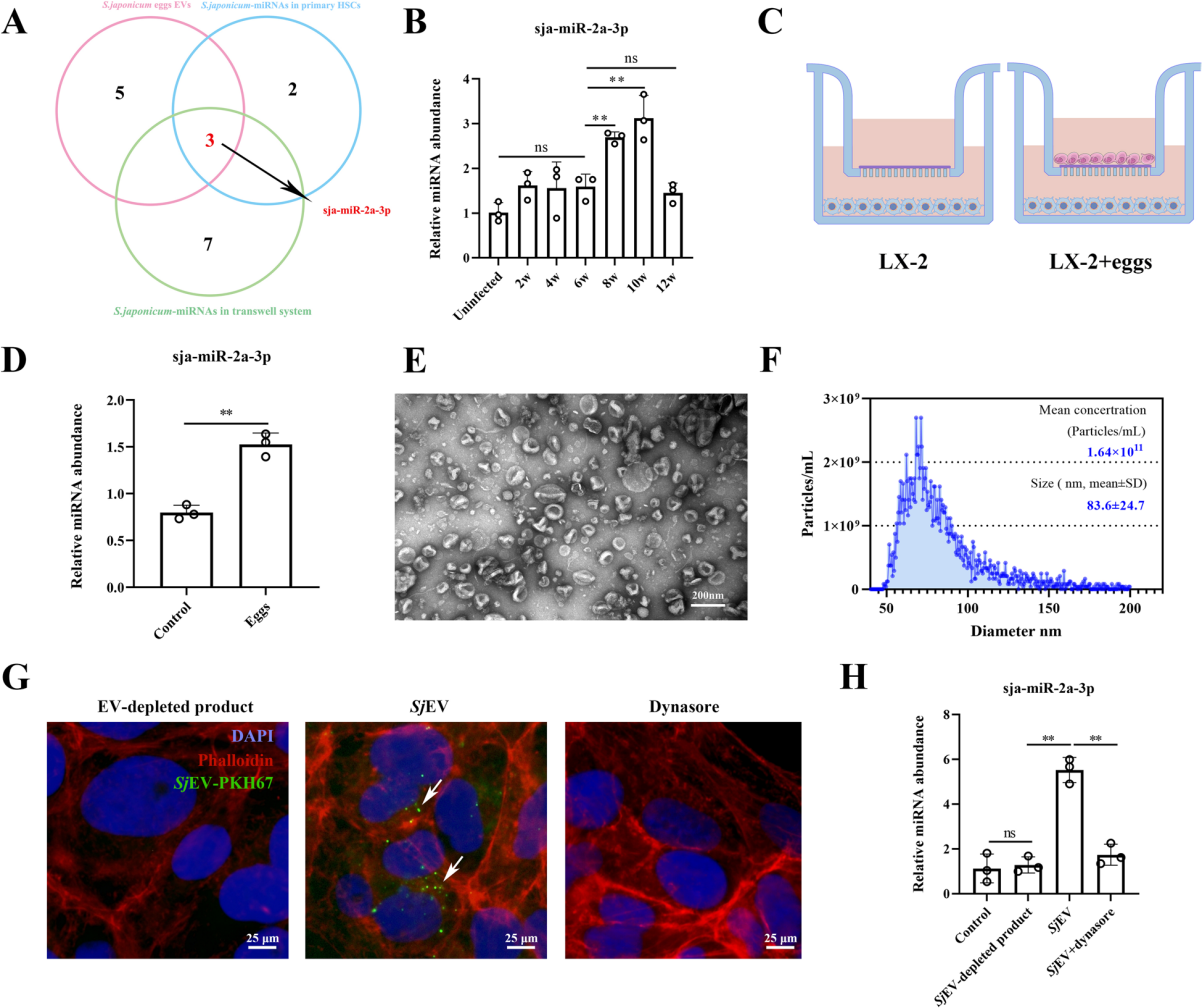
**sja-miR-2a-3p is delivered into HSCs via SjEVs.** **A** Venn diagram showing overlapping *Sj*-miRNAs identified in *S. japonicum* egg-derived EVs, primary HSCs from infected mice, and the transwell co-culture system. sja-miR-2a-3p was among the shared miRNAs. **B** Temporal expression of sja-miR-2a-3p in mouse liver tissues during *S. japonicum* infection at different time points (0–12 wpi) as determined by qPCR. Data are presented as mean ± SD (*n* = 3). **C** Schematic illustration of the transwell co-culture system in which *S. japonicum* eggs were placed in the upper chamber and HSCs in the lower chamber. **D** Relative abundance of sja-miR-2a-3p in HSCs after co-culture with eggs or control medium for 48 h (*n* = 3). **E** TEM image showing the morphology of isolated *Sj*EVs displaying typical cup-shaped vesicles (scale bar = 200 nm). **F** NTA of *Sj*EVs showing particle size distribution and concentration. **G** Representative fluorescence images of LX-2 cells incubated with PKH67-labeled *Sj*EVs, *Sj*EV-depleted products, or *Sj*EVs plus dynasore. Green signals indicate internalized *Sj*EVs; F-actin was stained with phalloidin (red) and nuclei with DAPI (blue) (scale bar = 25 μm). **H** qPCR analysis of sja-miR-2a-3p abundance in HSCs treated with dimethyl sulfoxide (DMSO), *Sj*EV-depleted products, *Sj*EVs, or *Sj*EVs plus dynasore (endocytosis inhibitor) (*n* = 3). All data are expressed as mean ± SD from three independent experiments. Statistical significance was determined using Student’s *t*-test where appropriate (^*^p < 0.05; ^**^p < 0.01., ns = not significant).

To explore whether schistosome-derived miRNAs are transferred into host HSCs, we compared Sj-miRNAs identified in egg-derived EVs [22], primary HSCs from infected mice [15], and our transwell co-culture system [19]. The Venn diagram (Figure 1A and Additional file 6) revealed three overlapping *Sj*-miRNAs, including sja-miR-2a-3p, indicating that this miRNA is likely delivered to HSCs via *Sj*EVs. Given its appearance among the shared *Sj*-miRNAs, sequence analysis across multiple helminth species was performed and demonstrated that miR-2a family members are highly conserved among helminth, suggesting an evolutionarily preserved regulatory function (Additional file 7). Analysis of a previously established infection time-course dataset[21] showed that hepatic sja-miR-2a-3p levels progressively increased during infection, peaked at 10 weeks post-infection (wpi), and slightly declined at 12 wpi (Figure 1B), indicating its potential involvement in fibrosis progression.

Previous work from our group using a worm–HSC co-culture system demonstrated that sja-miR-2a-3p could be detected in HSCs, indicating that parasite-associated sja-miR-2a-3p can be transferred to host cells under co-culture conditions. In the present study, to further assess whether a similar transfer could also occur in an egg-related system, we established a transwell model in which *S. japonicum* eggs were co-cultured with LX-2 cells without direct contact (Figure 1C). After 48 h, sja-miR-2a-3p abundance was significantly increased in LX-2 cells compared with controls (Figure 1D), supporting egg-to-HSC miRNA transfer.

*Sj*EVs were then isolated and characterized. TEM revealed typical cup-shaped vesicles (Figure 1E), and NTA showed a mean diameter of 83.6 ± 24.7 nm with a concentration of 1.64 × 10^11^ particles/mL (Figure 1F), consistent with EV characteristics and no vesicles were detected in the depleted product (Additional file 8). Proteomic profiling confirmed the presence of characteristic EV-associated proteins, including HSP60, HSP70, HSP90, GAPDH, CD63, annexin, actin, and tubulin (Additional files 9 and 10). PKH67 labeling demonstrated efficient uptake of *Sj*EVs by LX-2 cells, whereas EV-depleted products showed no signal (Figure 1G). Correspondingly, exposure to *Sj*EVs significantly increased intracellular sja-miR-2a-3p levels, while EV-depleted products had no effect. Importantly, pretreatment with the endocytosis inhibitor dynasore abolished both EV uptake and the elevation of sja-miR-2a-3p (Figure 1H). Together, these findings demonstrate that sja-miR-2a-3p can be delivered into HSCs via *Sj*EVs.

### sja-miR-2a-3p directly targets BCL2

To investigate the functional role of sja-miR-2a-3p, potential host target genes were predicted and subjected to pathway enrichment analysis. Predicted targets were significantly enriched in apoptosis-, inflammation-, and mitochondria-related pathways (Additional files 11 and 12), suggesting a role in mitochondrial apoptotic regulation. Among these candidates, the anti-apoptotic gene BCL2 was identified as a putative direct target.

**Figure 2:**
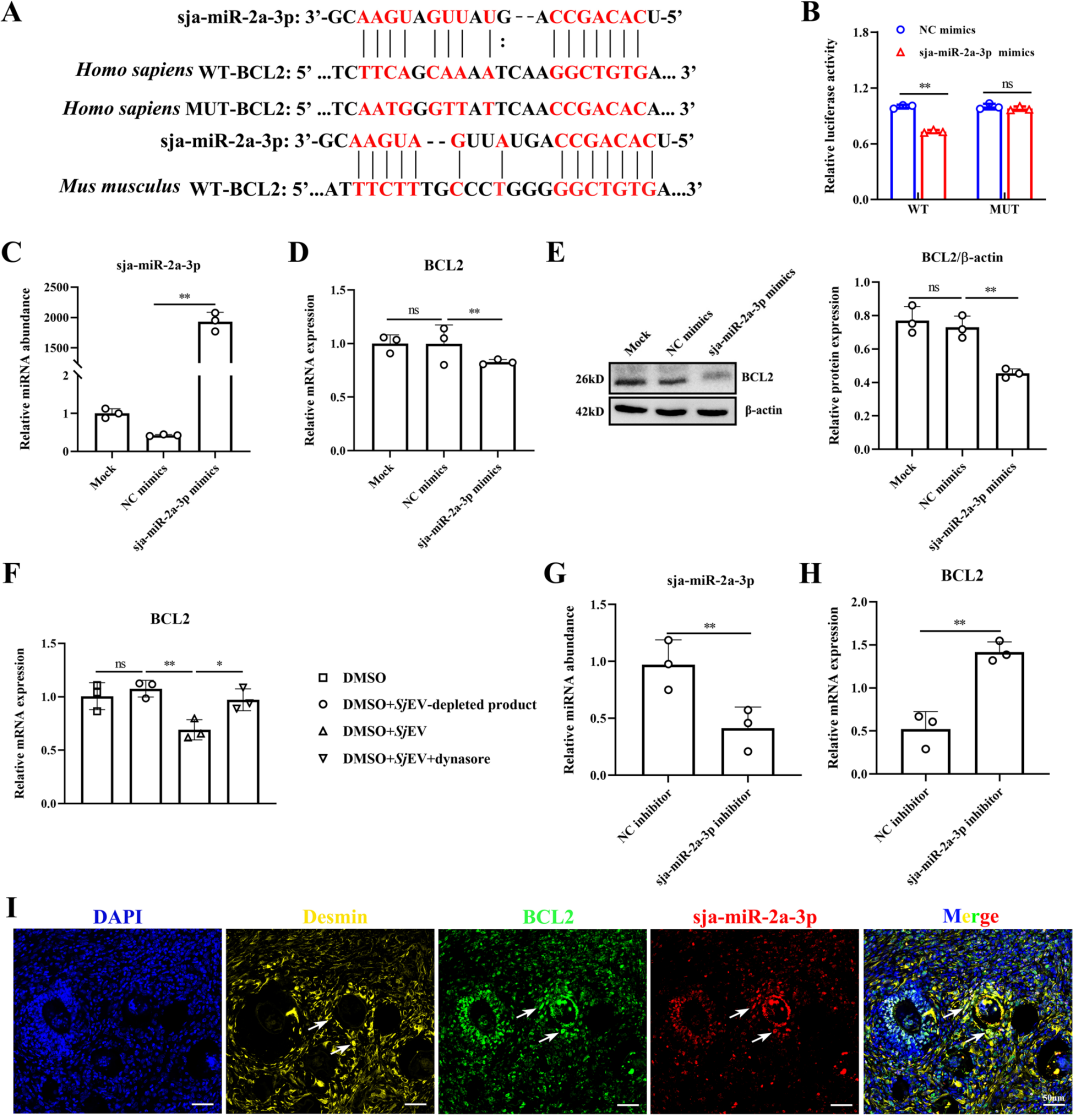
**sja-miR-2a-3p directly targets and suppresses BCL2 expression.** **A** Sequence alignment showing the complementary binding of sja-miR-2a-3p to the wild-type (WT-BCL2) 3′UTR of mouse BCL2 and the mutated sequence (MUT-BCL2) disrupting the binding site. **B** Dual-luciferase reporter assay (*n* = 3). **C** qPCR analysis showing significantly elevated levels of sja-miR-2a-3p in LX-2 cells transfected with sja-miR-2a-3p mimics (*n* = 3). **D**–**E** mRNA (**D**) and protein (**E**, representative western blot and quantification) levels of BCL2 are significantly reduced in the sja-miR-2a-3p mimics group (*n* = 3). **F** Treatment with *Sj*EVs significantly reduces BCL2 mRNA expression compared with DMSO or *Sj*EV-depleted product, while this effect is abolished by the endocytosis inhibitor dynasore (*n* = 3). **G**–**H** Transfection with sja-miR-2a-3p inhibitor in *Sj*EVs-treated LX-2 cells decreases sja-miR-2a-3p levels (**G**) and restores BCL2 mRNA expression (H) (*n* = 3). **I** Double FISH combined with immunofluorescence in mouse liver sections: Co-localization of sja-miR-2a-3p (red) and BCL2 (green) in desmin-positive (yellow) hepatic stellate cells (white arrows); DAPI (blue) stains the nuclei (scale bar = 50 μm). Data are presented as mean ± SD. Statistical analysis was performed using Student’s *t*-test as appropriate; ns, not significant (^*^p < 0.05; ^**^p < 0.01., ns = not significant).

Sequence analysis revealed a complementary binding site for sja-miR-2a-3p within the 3′UTR of BCL2 (Figure 2A). Dual-luciferase assays confirmed this interaction, as sja-miR-2a-3p significantly reduced luciferase activity of the wild-type BCL2 reporter but not the mutant construct (Figure 2B). In LX-2 cells, overexpression of sja-miR-2a-3p markedly decreased both BCL2 mRNA and protein levels (Figure 2C–E).

To further evaluate downstream effects, RNA-sequencing (RNA-seq) analysis was performed on LX-2 cells transfected with NC or sja-miR-2a-3p mimics. Principal component analysis indicated clear sample separation, and differential expression analysis identified 610 downregulated and 613 upregulated genes (Additional file 13). RNA-seq analysis further demonstrated broad transcriptional changes following sja-miR-2a-3p overexpression, with enrichment in apoptosis-related signaling pathways, including PI3K–Akt, MAPK, p53, and oxidative phosphorylation pathways (Additional file 14), supporting its role in mitochondrial homeostasis and cell death regulation. Although BCL2 did not reach statistical significance in RNA-seq, a consistent downward trend was observed (Additional file 15), consistent with qPCR validation. Notably, our previous egg–LX-2 co-culture dataset also showed reduced BCL2 expression (Additional file 15), implicating schistosome-derived factors in its regulation.

In the EV co-culture model, *Sj*EV exposure significantly suppressed BCL2 expression in HSCs, whereas dynasore treatment abolished this effect (Figure 2F). Conversely, inhibition of sja-miR-2a-3p restored BCL2 levels following *Sj*EV stimulation (Figure 2G–H), confirming that BCL2 downregulation is mediated by EV-delivered sja-miR-2a-3p.

In vivo, dual FISH combined with desmin immunostaining demonstrated co-localization of sja-miR-2a-3p and BCL2 within HSCs in fibrotic liver tissues (Figure 2I), supporting their functional interaction in the disease context.

Collectively, these findings demonstrate that sja-miR-2a-3p directly targets and suppresses host BCL2, linking schistosome-derived miRNA delivery to mitochondrial apoptotic regulation in HSCs.

### sja-miR-2a-3p triggers mitochondrial pathway-mediated apoptosis of HSCs in vitro

**Figure 3:**
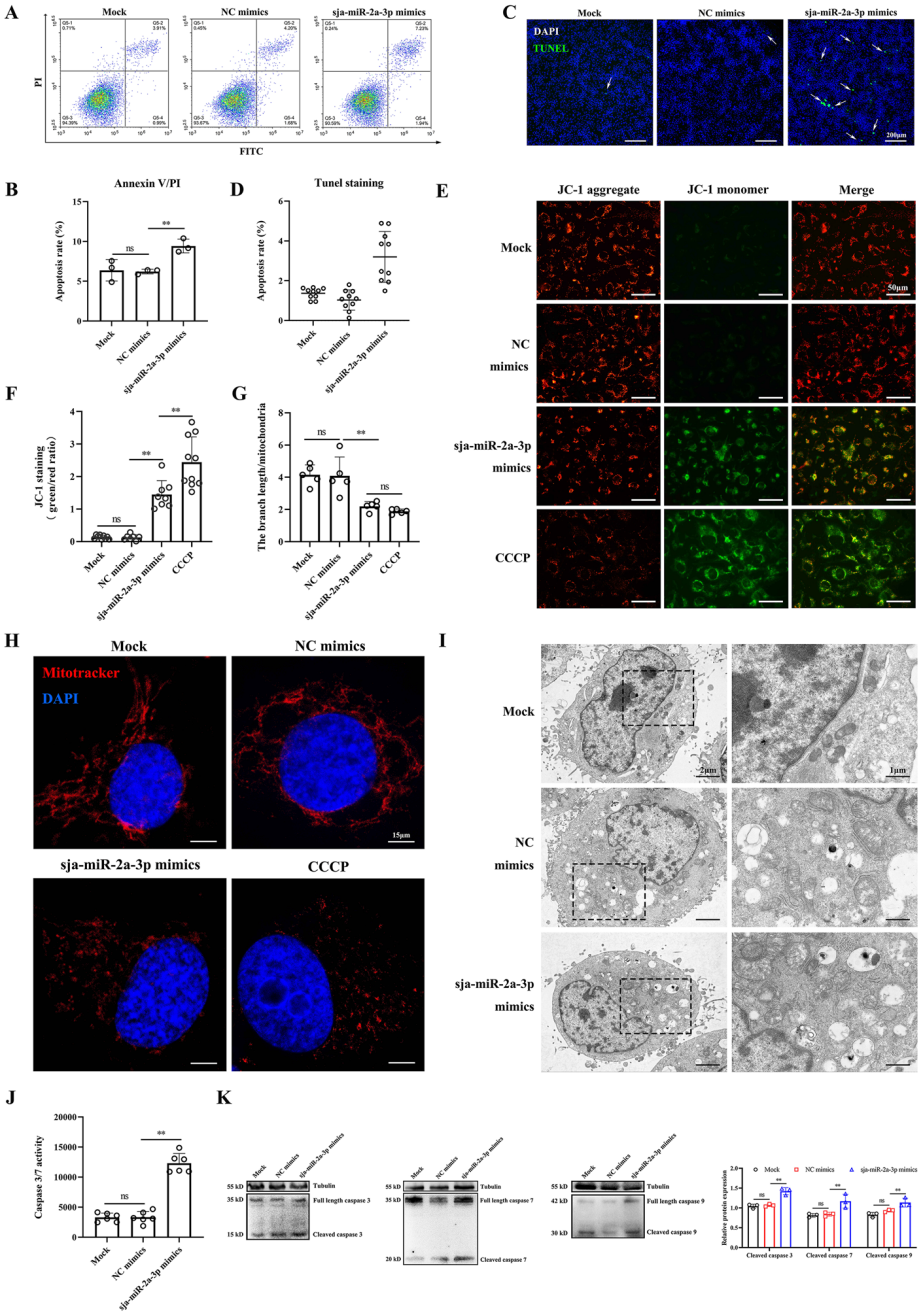
**sja-miR-2a-3p induces apoptosis and mitochondrial dysfunction in LX-2 cells.** **A** Representative flow cytometry analysis showing apoptosis in LX-2 cells transfected with sja-miR-2a-3p mimics compared with mock and NC mimics groups. **B** Quantification of apoptosis rate based on flow cytometry analysis (*n* = 3). **C** TUNEL staining showing apoptotic cells in LX-2 cells transfected with sja-miR-2a-3p mimics (green staining) compared with mock and NC mimics groups. DAPI (blue) marks the nuclei. **D** Quantification of TUNEL-positive cells in each group (*n* = 10). **E** JC-1 staining showing mitochondrial membrane potential: JC-1 aggregates (red) indicate healthy mitochondria, while JC-1 monomers (green) indicate depolarized mitochondria. The loss of red fluorescence and increase in green fluorescence indicates mitochondrial depolarization in sja-miR-2a-3p mimics-treated cells. **F** Quantification of JC-1 staining showing significant mitochondrial depolarization in sja-miR-2a-3p mimics-treated cells (*n* = 10). **G** Mitochondrial morphology analysis using MitoTracker staining (red) and DAPI (blue) showing changes in mitochondrial structure after sja-miR-2a-3p treatment (*n* = 5). **H** Enlargement of mitochondrial images showing detailed ultrastructural changes (dashed boxes indicate areas magnified on the right). **I** Representative TEM images of LX-2 cells showing mitochondrial fragmentation and swelling in the sja-miR-2a-3p mimics group (arrows). **J** Caspase-3/7 activity assay indicating activation of apoptosis in sja-miR-2a-3p mimics-treated cells compared with mock and NC mimics groups (*n* = 6). **K** Western blot analysis showing cleavage of caspase-3, caspase-7, and caspase-9 in LX-2 cells treated with sja-miR-2a-3p mimics. Tubulin is used as a loading control. Quantification of cleaved caspase-3, caspase-7, and caspase-9 bands is shown (*n* = 3). Data are presented as mean ± SD. Statistical analysis was performed using Student’s *t*-test as appropriate (^*^p < 0.05; ^**^p < 0.01., ns = not significant).

Since BCL2 is a key anti-apoptotic protein located on the mitochondrial outer membrane, the mitochondrial apoptotic pathway was examined to determine whether it was affected by sja-miR-2a-3p. Apoptosis was assessed by Annexin V/PI flow cytometry, which revealed a significant increase in apoptotic cells after transfection with sja-miR-2a-3p mimics compared with the mock and NC groups (Figure 3A and B). Enhanced DNA fragmentation was further confirmed by TUNEL staining, in which more TUNEL-positive nuclei were observed in the sja-miR-2a-3p mimic-treated cells (Figure 3C and D).

To assess mitochondrial involvement, the mitochondrial membrane potential was examined by JC-1 staining, which revealed a marked loss of red fluorescence and an increased green signal, indicating depolarization of the mitochondrial membrane potential in sja-miR-2a-3p mimic-transfected cells, similar to that observed in the CCCP-treated group (Figure 3E and F). Consistently, MitoTracker staining showed extensive mitochondrial fragmentation and shortened mitochondrial branches (Figure 3G and H). These findings were supported by TEM, which revealed swollen mitochondria and disrupted cristae in the sja-miR-2a-3p mimic group (Figure 3I).

To further confirm activation of the mitochondrial apoptotic cascade, caspase activity and expression were analyzed. A significant increase in caspase-3 activity was detected in the sja-miR-2a-3p mimic group (Figure 3J). Western blot analysis further revealed marked cleavage of caspase-9, caspase-3, and caspase-7 (Figure 3K). Notably, activation of caspase-9, a key initiator caspase in the mitochondrial pathway, indicates that the observed apoptosis was mediated predominantly through the intrinsic, mitochondria-dependent signaling cascade.

Collectively, these findings indicate that sja-miR-2a-3p, through suppression of the mitochondrial anti-apoptotic molecule BCL2, induces mitochondrial dysfunction and activates the intrinsic, caspase-dependent apoptotic pathway in HSCs in vitro.

### Overexpression of sja-miR-2a-3p suppresses HSCs proliferation and migration

**Figure 4:**
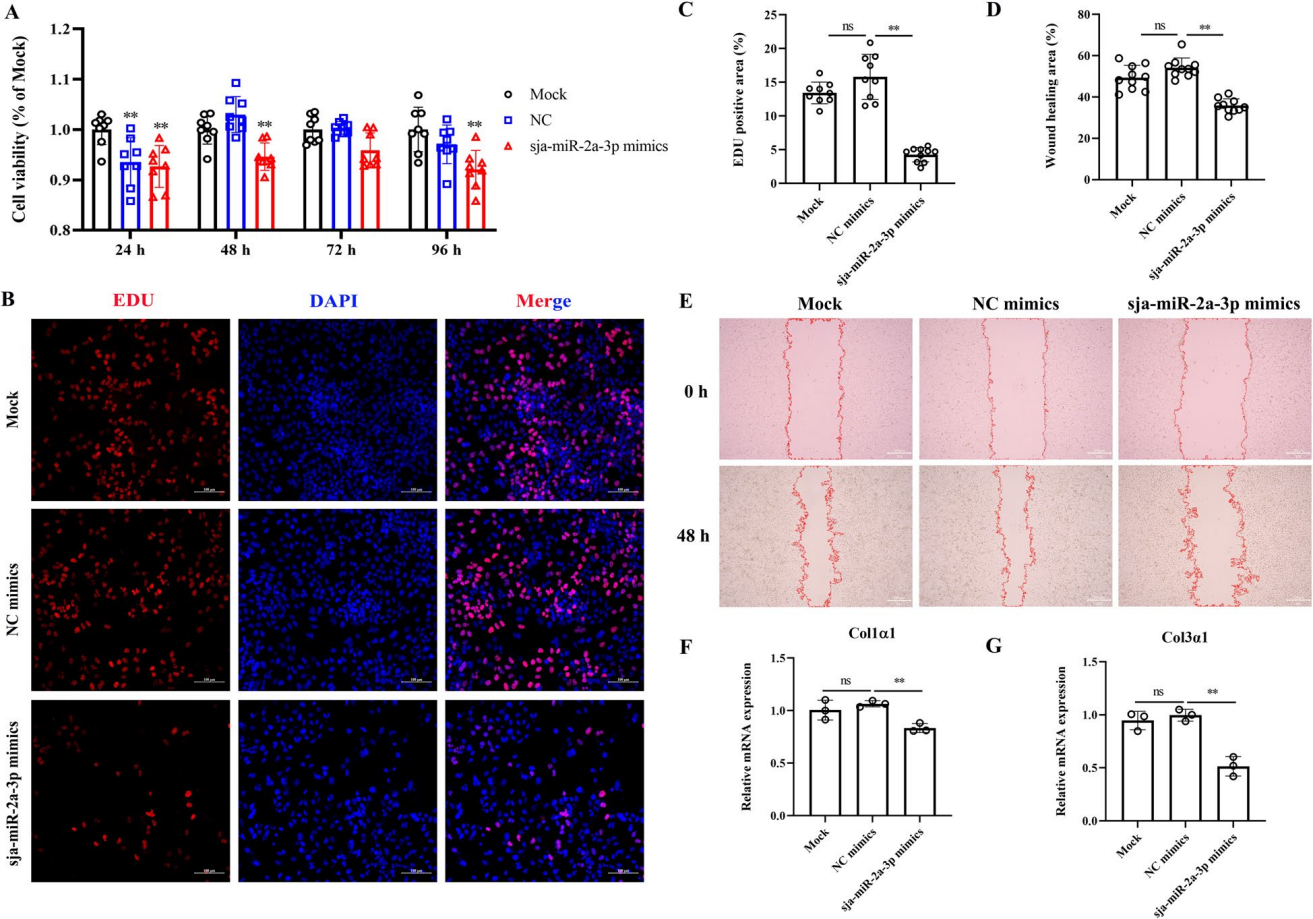
**Overexpression of sja-miR-2a-3p suppresses HSC proliferation and migration.** **A** Cell viability of LX-2 cells transfected with mock, NC mimics, or sja-miR-2a-3p mimics at 24, 48, 72, and 96 h, measured by CCK-8 assay (*n* = 8). **B** Representative fluorescence images of EdU incorporation (red) and DAPI nuclear staining (blue) in LX-2 cells. Merged images are shown (scale bar = 100 μm). **C** Quantification of EdU-positive cells (*n* = 9). **D** Quantitative analysis of wound healing area at 48 h (*n* = 10). **E** Representative images of wound-healing assays at 0 h and 48 h (scale bar = 200 μm). **F**–**G** Relative mRNA expression levels of Col1α1 (**F**) and Col3α1 (**G**) in LX-2 cells following transfection (*n* = 3). Data are presented as mean ± SD. Statistical analysis was performed using Student’s *t*-test as appropriate (^*^p < 0.05; ^**^p < 0.01., ns = not significant).

Given that sja-miR-2a-3p promotes mitochondrial apoptosis in HSCs, we next investigated whether its overexpression also affects HSC proliferative and migratory capacities, which are critical determinants of fibrogenic progression. CCK-8 analysis showed that overexpression of sja-miR-2a-3p significantly reduced cell viability at 24, 48, 72, and 96 h compared with mock and NC groups (Figure 4A), indicating suppressed proliferative capacity. Consistently, EdU incorporation assays demonstrated a marked decrease in the percentage of EdU-positive cells in the sja-miR-2a-3p mimics group (Figure 4B–C), further confirming inhibition of DNA synthesis and cell proliferation. Wound-healing assays revealed that sja-miR-2a-3p overexpression significantly impaired migratory ability, as evidenced by reduced wound closure at 48 h (Figure 4D–E). Quantitative analysis confirmed a significantly lower migration rate compared with controls. In addition, qPCR analysis showed that sja-miR-2a-3p overexpression significantly downregulated the mRNA expression of fibrogenic markers Col1α1 and Col3α1 (Figure 4F–G), suggesting that sja-miR-2a-3p not only suppresses HSC proliferation and migration but also attenuates extracellular matrix gene expression.

Collectively, these findings demonstrate that elevated sja-miR-2a-3p suppresses HSC proliferation, migration, and profibrotic gene expression, effects that are consistent with its pro-apoptotic activity and further contribute to the attenuation of fibrogenic progression.

### sja-miR-2a-3p induces HSC apoptosis and fibrosis resolution through suppression of BCL2

**Figure 5:**
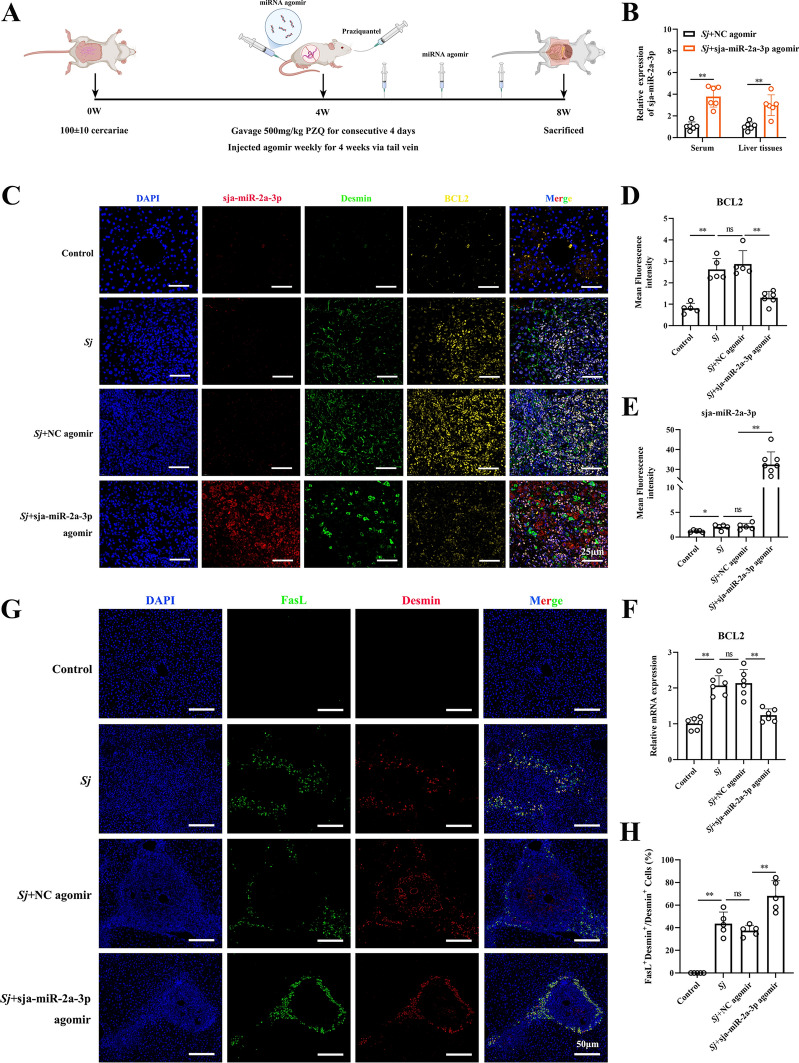
**sja-miR-2a-3p downregulates BCL2 and promotes HSC apoptosis in** ***S. japonicum*** **-infected mice.** **A** Experimental design of the *S. japonicum* infection and treatment model. BALB/c mice were percutaneously infected with 100 ± 10 cercariae and treated with PZQ (500 mg/kg, oral gavage for four consecutive days) at 4 wpi. Mice then received weekly tail-vein injections of NC agomir or sja-miR-2a-3p agomir for 4 weeks before sacrifice at 8 wpi. **B** qPCR quantification showing elevated sja-miR-2a-3p expression in serum and liver tissues of *S. japonicum*-infected mice following agomir administration compared with the NC agomir group (*n* ≥ 5). **C** Representative double FISH and immunofluorescence images showing co-localization of sja-miR-2a-3p (red) and BCL2 (yellow) within desmin-positive HSCs (green) in mouse liver sections. DAPI (blue) marks nuclei; scale bar = 25 μm. **D**–**E** Quantification of BCL2 (**D**) and sja-miR-2a-3p (**E**) fluorescence intensity (*n* ≥ 5). **F** qPCR analysis of hepatic BCL2 mRNA levels. **G** Dual immunofluorescence staining of FasL (green) and desmin (red) in mouse liver sections. Scale bar = 50 μm (*n* ≥ 5). **H** Quantitative analysis of FasL⁺/desmin⁺ cell proportion showing significantly increased apoptosis in sja-miR-2a-3p agomir-treated mice (*n* ≥ 5). Data are expressed as mean ± SD. Statistical analysis was performed using Student’s *t*-test as appropriate (^*^p < 0.05; ^**^p < 0.01., ns = not significant). Panel A was created with Biorender.

To determine whether sja-miR-2a-3p exerts similar regulatory effects in vivo, *S. japonicum*-infected mice were treated with sja-miR-2a-3p agomir following praziquantel (PZQ) administration as shown in Figure 5A. PZQ was administered to eliminate adult worms and thereby synchronize egg deposition in the liver, ensuring that fibrosis development was driven by eggs of comparable developmental stages and minimizing variability caused by asynchronous egg maturation. This strategy allowed a more controlled assessment of the antifibrotic effect of sja-miR-2a-3p. qPCR analysis confirmed a significant elevation of sja-miR-2a-3p expression in both serum and liver tissues after agomir treatment compared with the NC agomir group (Figure 5B), indicating successful delivery of the miRNA.

Dual FISH and immunofluorescence staining were used to examine the localization of sja-miR-2a-3p and its target BCL2 in HSCs. Co-localization of sja-miR-2a-3p (red) and BCL2 (yellow) within desmin-positive HSCs (green) was observed in infected liver tissues (Figure 5C). Quantitative analysis revealed that BCL2 fluorescence intensity was markedly decreased in the sja-miR-2a-3p agomir group (Figure 5D), whereas sja-miR-2a-3p signal intensity was significantly increased (Figure 5E), demonstrating an inverse correlation between the schistosome-derived miRNA and its host target in vivo. To further validate these findings, BCL2 mRNA levels were measured by qPCR and found to be significantly downregulated in the sja-miR-2a-3p agomir group compared with the *S. japonicum*-infected and NC-agomir groups (Figure 5F). Moreover, dual immunofluorescence staining for FasL (a apoptotic marker) and desmin revealed a higher proportion of FasL⁺/desmin⁺ cells in sja-miR-2a-3p agomir-treated mice (Figure 5G and H), indicating enhanced apoptosis of HSCs following BCL2 suppression.

Collectively, these results demonstrate that sja-miR-2a-3p, delivered via agomir in *S. japonicum*-infected mice, effectively downregulates the anti-apoptotic gene BCL2 and promotes apoptosis in HSCs, providing in vivo evidence that this schistosome-derived miRNA contributes to fibrosis resolution by modulating host apoptotic signaling.

### Elevating sja-miR-2a-3p levels alleviates liver fibrosis and inflammation in *S. japonicum*-infected mice

**Figure 6:**
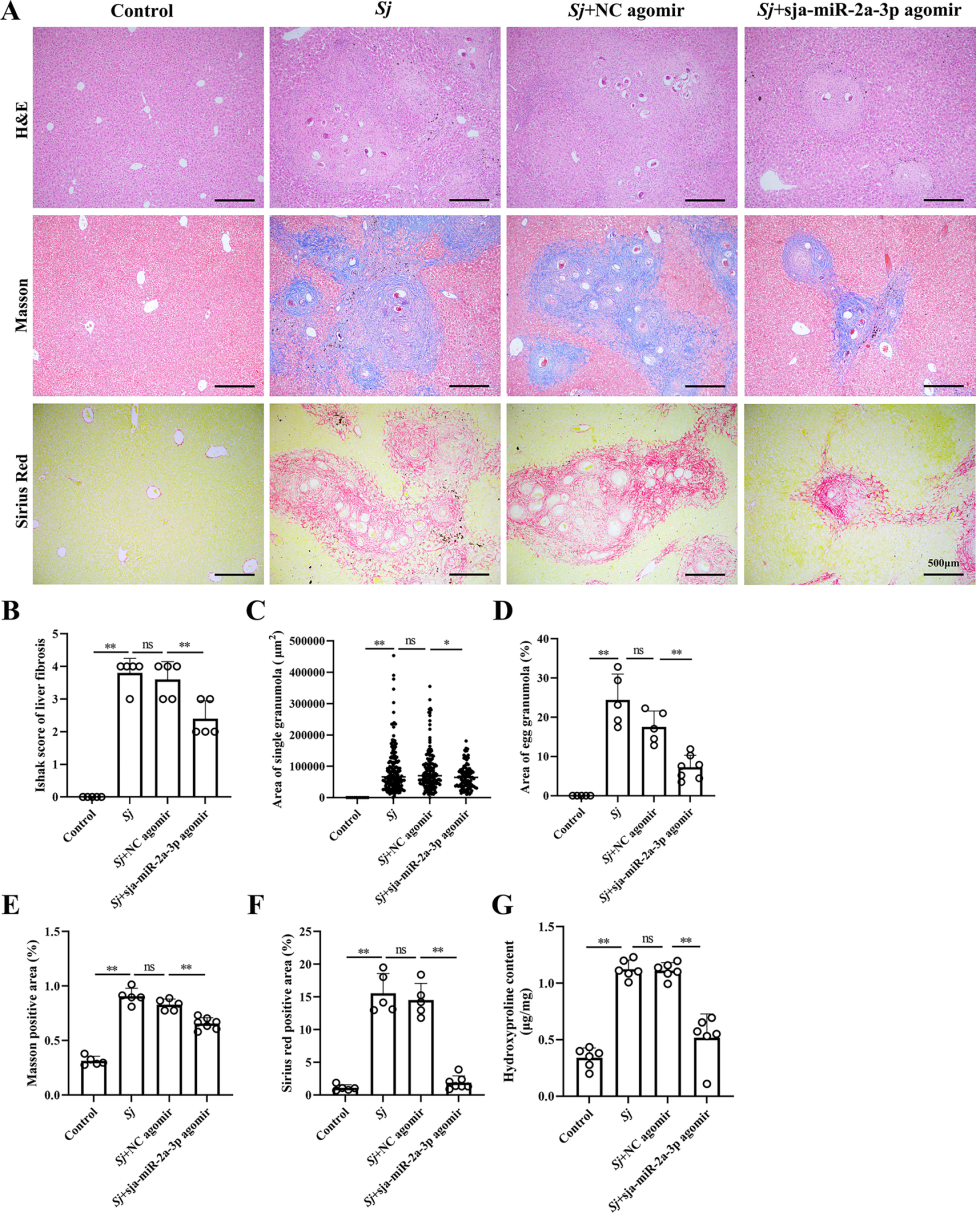
**Histological analysis and quantification of liver fibrosis in** ***S. japonicum*** **-infected mice treated with sja-miR-2a-3p agomir.** **A** Representative histological images of liver sections from different treatment groups stained with H&E, Masson’s trichrome, and Sirius Red. Scale bar = 500 μm. **B** Ishak fibrosis score in liver tissues of each group (*n* ≥ 5). **C** Quantification of the area of single egg granulomas in liver sections. **D** Proportion of granuloma area in the liver (*n* ≥ 5). **E** Masson’s trichrome positive area analysis (*n* ≥ 5). **F** Sirius Red positive area analysis (*n* ≥ 5). **G** Hydroxyproline content in liver tissues (*n* ≥ 5). Data are expressed as mean ± SD. Statistical analysis was performed using Student’s *t*-test as appropriate (^*^p < 0.05; ^**^p < 0.01., ns = not significant).

To determine whether sja-miR-2a-3p–induced HSC apoptosis attenuates liver fibrosis in vivo, histological analyses were performed. Compared with infected and NC-agomir groups, sja-miR-2a-3p-treated mice exhibited markedly reduced granuloma formation, collagen deposition, and fibrosis severity, as shown by H&E, Masson, and Sirius Red staining (Figure 6A). Quantitative analysis confirmed significant decreases in Ishak fibrosis scores, granuloma size and area, collagen-positive staining, and hepatic hydroxyproline content (Figure 6B–G).

**Figure 7:**
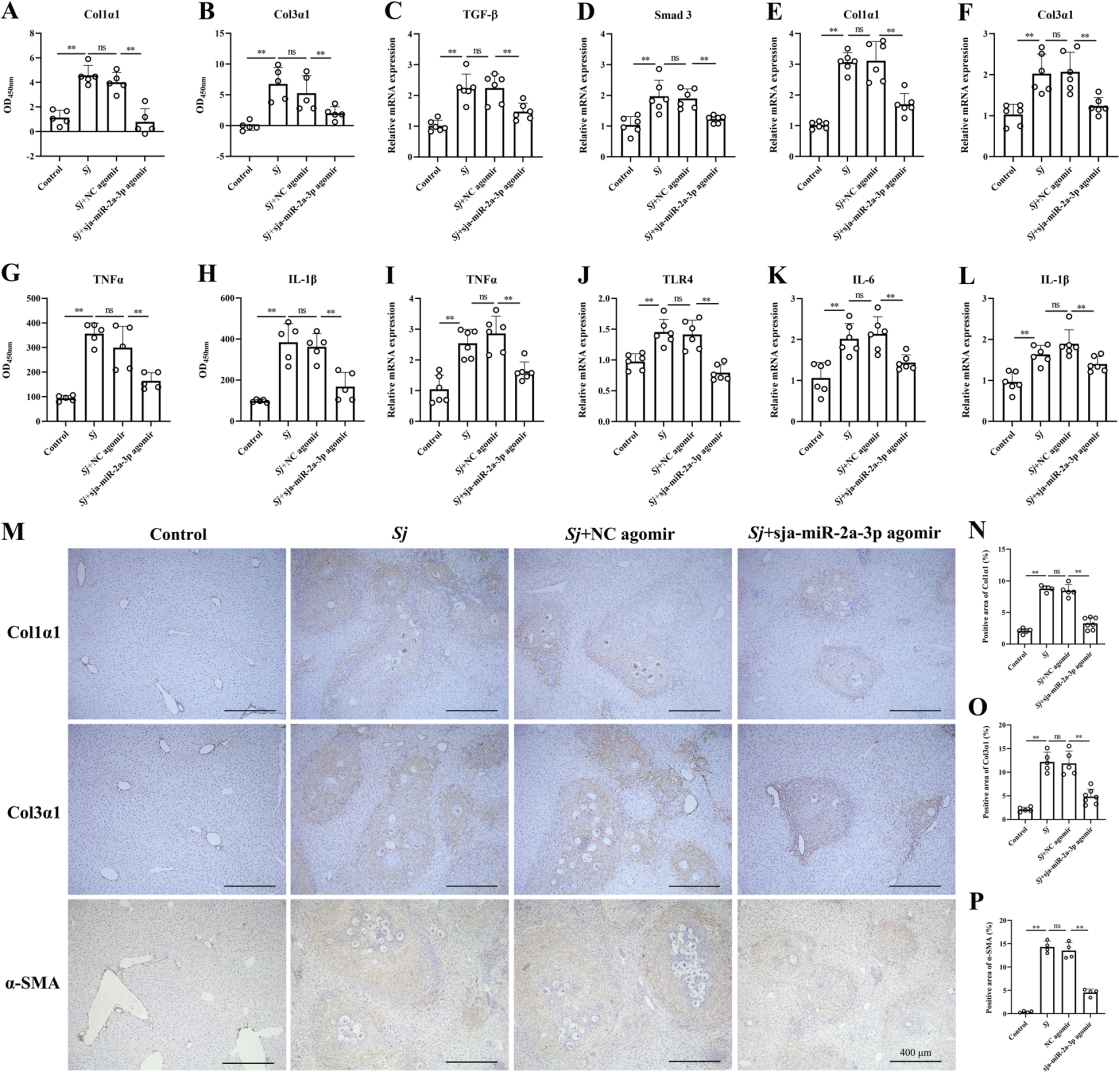
**sja-miR-2a-3p treatment alleviates hepatic fibrosis and inflammation in** ***S. japonicum*** **-infected mice.** **A**–**B** ELISA quantification of Col1α1 and Col3α1 protein levels in serum from each group (*n* ≥ 5). **C**–**F** qPCR analysis showing relative hepatic mRNA expression of TGF-β, Smad3, Col1α1, and Col3α1 (*n* ≥ 5). **G**–**H** ELISA analysis of serum TNF-α and IL-1β protein levels (*n* ≥ 5). **I**–**L** qPCR analysis showing hepatic mRNA expression of TNF-α, TLR4, IL-6, and IL-1β (*n* ≥ 5). **M** Representative immunohistochemical staining of Col1α1, Col3α1, and α-SMA in liver sections. Scale bar = 400 μm. **N**–**P** Quantification of immunohistochemical positive staining areas for Col1α1, Col3α1, and α-SMA, respectively. Data are expressed as mean ± SD. Statistical analysis was performed using Student’s *t*-test as appropriate (^*^p < 0.05; ^**^p < 0.01., ns = not significant).

At the molecular level, serum enzyme-linked immunosorbent assay (ELISA) showed reduced levels of Col1α1, Col3α1, TNF-α, and IL-1β (Figure 7A, B, G, H). Consistently, qPCR revealed downregulation of fibrogenic genes (TGF-β, Smad3, Col1α1, Col3α1) and inflammatory mediators (TNF-α, TLR4, IL-6, IL-1β) (Figure 7C–L). Immunohistochemistry further confirmed decreased hepatic expression of Col1α1, Col3α1, and α-SMA (Figure 7M–P).

Peripheral blood analysis demonstrated that sja-miR-2a-3p treatment partially reversed infection-induced increases in total white blood cells and neutrophils (Additional file 16), indicating attenuation of systemic inflammation. Importantly, histological examination of major organs revealed no detectable toxicity following agomir administration (Additional file 17).

Collectively, these results demonstrate that elevating sja-miR-2a-3p effectively alleviates hepatic fibrosis and inflammation while exhibiting favorable in vivo safety, supporting its therapeutic potential.

## Discussion

**Figure 8:**
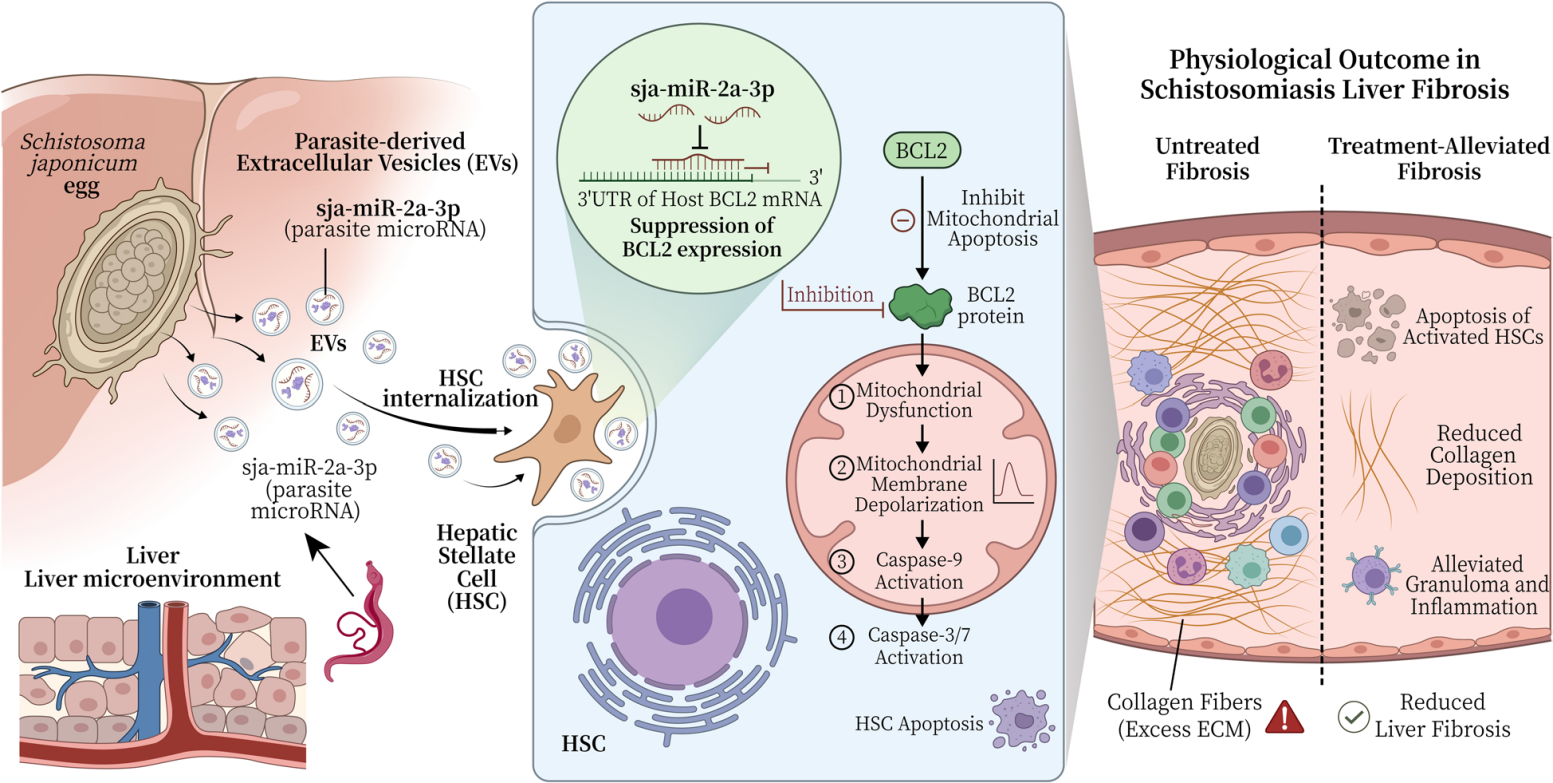
**Graphical abstract of the mechanism.** During *S. japonicum* infection, EVs released by the parasite are taken up by HSCs in the fibrotic liver. These EVs deliver sja-miR-2a-3p into HSCs, where it binds to the (3′UTR) of the host anti-apoptotic gene BCL2, leading to mRNA degradation and suppression of BCL2 protein expression. The resulting inhibition of BCL2 activates the mitochondrial apoptosis pathway, promoting HSC apoptosis. This process reduces the population of activated HSCs and contributes to the attenuation of schistosome-induced liver fibrosis. This figure was created with Biorender.

The present study demonstrates that helminth-specific sja-miR-2a-3p, delivered by *Sj*EVs, plays a pivotal regulatory role in schistosome-induced hepatic fibrosis by promoting apoptosis of HSCs. sja-miR-2a-3p functions as an important effector molecule that targets host BCL2, thereby activating the mitochondrial apoptosis pathway in HSCs. This process reduces the number of activated HSCs and limits excessive extracellular matrix deposition, ultimately attenuating hepatic fibrosis during schistosome infection. These findings reveal a previously unrecognized mechanism through which parasite-derived regulatory molecules influence host pathological responses (Figure 8).

*S. japonicum* is a zoonotic parasite maintained in a broad range of mammalian hosts, including livestock and other animal reservoirs that contribute to transmission in endemic areas [3]. Chronic hepatointestinal schistosomiasis is therefore not only a human health problem but also a disease of veterinary and One Health importance [4]. Hepatic fibrosis is one of the most important pathological consequences of chronic *S. japonicum* infection, reflecting sustained host responses to parasite eggs trapped in the liver [37]. Clarifying how schistosome-derived molecules regulate this process may improve our understanding of schistosome-associated pathology across zoonotic host systems and may help explain how chronic infection is established and maintained.

Hepatic fibrosis fundamentally arises from the fate transitions of HSCs, encompassing their persistent activation with excessive extracellular matrix deposition, reversion to a quiescent state, or clearance through programmed cell death pathways [38]. Among these mechanisms, HSC apoptosis is considered one of the crucial factors of fibrosis regression, as it effectively decreases the population of activated cells and facilitates the degradation of extracellular matrix components [39]. Recent studies have highlighted that pharmacological induction of HSC apoptosis can significantly attenuate liver fibrosis. For instance, tenofovir disoproxil fumarate reduces collagen accumulation and improves fibrosis by inducing HSC apoptosis through inhibition of the PI3K/Akt/mTOR signaling pathway [40]. Dihydroartemisinin enhances Bax expression, suppresses BCL2, and activates the caspase cascade, ultimately triggering apoptosis and reducing fibrotic tissue formation [41]. Carvedilol, widely used in cardiovascular therapy, has also been reported to induce apoptosis in HSCs via activation of caspase-3, caspase-8, and PARP cleavage [42]. Collectively, these findings indicate that clearance of activated HSCs through apoptosis represents a promising route for fibrosis reversal.

During long-term host–parasite interaction, *Schistosoma* species may mitigate host pathology to maintain a balanced parasitic niche and prolong their survival [19]. EVs secreted by the schistosome have been recognized as pivotal mediators of this communication. Egg-derived EVs release sja-miR-71a, which alleviates HSC activation and hepatic fibrosis by inhibiting the TGF-β/Smad signaling pathway [14]; worm-derived EVs deliver sja-let-7, targeting Col1α2 to reduce type I collagen synthesis and fibrosis severity [21]. These observations suggest that schistosomes employ both immunomodulatory and molecular strategies—such as EV-mediated miRNA transfer—to minimize excessive tissue damage while sustaining chronic infection.

Earlier studies suggested that sja-miR-2a-3p may play a regulatory role in HSC activation [15, 19]. In addition, previous work identified sja-miR-2a-3p as an abundantly expressed miRNA in *S. japonicum* worms from non-permissive hosts, where parasites often exhibit developmental arrest accompanied by apoptotic features. sja-miR-2a-3p is predicted to target a *S. japonicum* BCL2-like apoptosis inhibitor [43], suggesting a potential association between sja-miR-2a-3p and apoptosis [44, 45]. Notably, miR-2a family members are highly conserved among parasitic helminths and have been detected in the serum of infected hosts, indicating that these miRNAs may participate in cross-species molecular communication during infection [46–52]. Collectively, these findings led to the hypothesis that sja-miR-2a-3p may be delivered into host HSCs through parasite-derived EVs, where it could target BCL2 to induce apoptosis and thereby modulate schistosomiasis-associated hepatic fibrosis.

In the present study, experiments designed to test this hypothesis revealed that schistosome EV-associated sja-miR-2a-3p entered host HSCs, suppressed BCL2 expression, and triggered the mitochondrial apoptosis pathway. In addition to its pro-apoptotic effect, sja-miR-2a-3p also suppressed HSC proliferation and migration in vitro. Overexpression of sja-miR-2a-3p significantly reduced cell viability, DNA synthesis, wound closure capacity, and the expression of profibrotic genes such as Col1α1 and Col3α1. These findings suggest that sja-miR-2a-3p not only promotes apoptotic clearance of activated HSCs but may also restrain their expansion and fibrogenic activity though the specific mechanisms still need to be discovered. Therefore, the antifibrotic effect of sja-miR-2a-3p is likely mediated through both apoptosis induction and functional suppression of HSC activation. Such dual regulation may enhance fibrosis resolution by limiting both the number and the activity of fibrogenic cells. Consistent with these cellular findings, administration of sja-miR-2a-3p agomir via tail vein injection significantly increased its hepatic abundance and attenuated fibrotic progression in vivo. Co-localization of desmin and FasL indicated enhanced apoptosis of HSCs, confirming that sja-miR-2a-3p exerts pro-apoptotic effects in the host.

Beyond its direct effect on BCL2, transcriptomic enrichment analysis indicated that sja-miR-2a-3p also modulates pathways involved in mitochondrial function, oxidative phosphorylation, and PI3K–Akt and MAPK signaling. This suggests a broader regulatory spectrum, possibly integrating both intrinsic and extrinsic apoptotic pathways. Furthermore, this controlled induction of HSC apoptosis may serve not only to limit fibrosis but also to maintain host–parasite homeostasis. By moderating excessive tissue injury, the parasite may create a host environment conducive to chronic infection while avoiding fatal damage to the host [18]. Such fine-tuned regulation exemplifies a parasite adaptation strategy that balances pathogenicity with persistence. Targeting the miRNA–BCL2 axis could also provide a novel antifibrotic approach distinct from conventional drugs acting on TGF-β or PI3K/Akt pathways.

Despite the promising findings, several limitations merit consideration. Although BCL2 was prioritized for mechanistic validation on the basis of dual-program target prediction and its clear relevance to mitochondrial apoptosis, other predicted target genes of sja-miR-2a-3p were not systematically examined in the present study. In addition, the predicted binding sites within the BCL2 3′UTR are conserved between human and mouse, but further comparative validation would strengthen the cross-species interpretation of these findings. In addition to its direct effect on HSCs, sja-miR-2a-3p may have broader effects on liver cell types. While this study focused on HSC apoptosis, it remains to be determined whether sja-miR-2a-3p affects hepatocytes, Kupffer cells, or other liver cells. In addition, although the FISH analysis in Figure 2I provided tissue-level spatial support for the association between sja-miR-2a-3p and BCL2 in desmin-positive cells, the complex cellular composition within granulomatous regions may still increase the risk of signal misinterpretation or false-positive identification. Therefore, more direct approaches, such as primary HSC isolation combined with PCR/qPCR-based validation, will be needed in future studies to further strengthen the cell-specific interpretation of these findings. It is plausible that sja-miR-2a-3p may impact the function of other hepatic cell populations, further contributing to the observed antifibrotic effects. Another important consideration is the delivery of sja-miR-2a-3p by *Sj*EVs. While our data demonstrate that sja-miR-2a-3p is delivered to HSCs via *Sj*EVs, it remains unclear whether adult *S. japonicum* worms also secrete sja-miR-2a-3p using or without using EVs. Further studies are needed to clarify the contribution of adult worms in the secretion and delivery of sja-miR-2a-3p and whether other parasite-derived factors also play a role in the modulation of liver fibrosis. In addition, although the direct injection of agomir did not cause detectable damage to other organs or tissues, systematic toxicological and safety assessments remain necessary. Moreover, given that HSCs physiologically serve as the main storage site for vitamin A in the liver [9], vitamin A-modified liposomes or related nanocarriers could be employed in future studies to achieve HSC-specific targeting and minimize potential off-target effects [53, 54]. Furthermore, validation in additional hepatic fibrosis models, such as the carbon tetrachloride-induced model [55], would help determine the broader applicability of these findings.

In conclusion, this study demonstrates that *Sj*EVs deliver the helminth-derived miRNA sja-miR-2a-3p into host hepatic stellate cells, where it suppresses BCL2 expression and activates mitochondrial apoptosis, thereby attenuating hepatic fibrosis. These findings provide new insight into how a zoonotic helminth shapes host tissue pathology through EV-mediated molecular communication and highlight schistosome-derived miRNAs as potential regulators of chronic liver lesions during schistosomiasis.

## Supplementary Information

**Additional file 1. The sequence of miRNA mimics, inhibitor and agomir.**

**Additional file 2. Details for SjEVs collection and function analysis.**

**Additional file 3. Antibodies used in the experiment.**

**Additional file 4. Probes used in the FISH analysis.**

**Additional file 5. Primers used in the experiment.**

**Additional file 6.* S.japonicum*-miRNA in egg-EVs, primary HSCs and transwell co-culture system.**

**Additional file 7. Sequence conservation analysis of miR-2a family members among representative invertebrate species.**

**Additional file 8. TEM image of SjEV depleted ESPs. Scale bar, 500 nm**

**Additional file 9.* S. japonicum*** **proteins identified in EVs by LC–MS/MS.**

**Additional file 10. Schistosome EV-associated proteins selected from all EVs proteins identified by LC–MS/MS**

**Additional file 11. Functional analysis of target genes of sja-miR-2a-3p.**

**Additional file 12.** **Potential target genes of sja-miR-2a-3p identified by RNAhybrid and miRanda**.

**Additional file 13. Overview of RNA-seq.** **A** Principal component analysis. **B** Volcano plot of DEGs.

**Additional file 14. Functional analysis of DEGs from RNA-seq.** **A**–**C** GO enrichment analysis of DEGs. **D** KEGG analysis of DEGs.

**Additional file 15. RNA-seq validation of BCL2 downregulation by sja-miR-2a-3p.** **A** RNA-seq data (CNP0008176) showing normalized TPM of BCL2 in LX-2 cells transfected with NC mimics or sja-miR-2a-3p mimics. **B** RNA-seq data (CNP0007747) showing TPM values of BCL2 in LX-2 cells co-cultured with *S. japonicum* eggs or control medium. Data are expressed as mean ± SD (*n* = 3). Statistical analysis was performed using Student’s *t*-test as appropriate (** *p* < 0.01).

**Additional file 16. Hematological analysis of S. japonicum-infected mice following sja-miR-2a-3p agomir treatment.** ***S. japonicum*-infected mice following sja-miR-2a-3p agomir treatment.** **A** Representative scatter plots of WBC populations obtained by flow cytometry, showing neutrophils (Neu, cyan), lymphocytes (Lym, green), monocytes (Mon, purple), eosinophils (Eos, red), and basophils (Bas, blue). **B** Total WBC counts and **C** neutrophil counts in peripheral blood. Data are expressed as mean ± SD (*n* = 6). Statistical analysis was performed using Student’s *t*-test as appropriate (** *p* < 0.01; ns = not significant).

**Additional file 17. Histopathological evaluation of major organs following systemic administration of sja-miR-2a-3p agomir.** Scale bar = 200 μm.

**Additional file 18. Original picture for the immunoblotting results.**

## Data Availability

The datasets presented in this study can be found in online repositories. The names of the repository/repositories and accession number can be found below: CNGB Sequence Archive of China National GeneBank DataBase, CNP0008176.

## References

[1] Buonfrate D, Ferrari TCA, Akim Adegnika A, Russell Stothard J, Gobbi FG (2025) Human schistosomiasis. Lancet 405 :658–670. 10.1016/S0140-6736(24)02814-9 39986748 10.1016/S0140-6736(24)02814-9

[2] Lo NC, Bezerra FSM, Colley DG, Fleming FM, Homeida M, Kabatereine N, Kabole FM, King CH, Mafe MA, Midzi N, Mutapi F, Mwanga JR, Ramzy RMR, Satrija F, Stothard JR, Traoré MS, Webster JP, Utzinger J, Zhou X-N, Danso-Appiah A, Eusebi P, Loker ES, Obonyo CO, Quansah R, Liang S, Vaillant M, Murad MH, Hagan P, Garba A (2022) Review of 2022 WHO guidelines on the control and elimination of schistosomiasis. Lancet Infect Dis 22:e327–e335. 10.1016/S1473-3099(22)00221-3 35594896 10.1016/S1473-3099(22)00221-3

[3] McManus DP, Gray DJ, Li Y, Feng Z, Williams GM, Stewart D, Rey-Ladino J, Ross AG (2010) Schistosomiasis in the People’s Republic of China: the era of the Three Gorges Dam. Clin Microbiol Rev 23:442–466. 10.1128/CMR.00044-09 20375361 10.1128/CMR.00044-09 PMC2863366

[4] Grover E, Paull S, Kechris K, Buchwald A, James K, Liu Y, Carlton EJ (2022) Predictors of bovine *Schistosoma japonicum* infection in rural Sichuan, China. Int J Parasitol 52:485–496. 10.1016/j.ijpara.2022.04.002 35644269 10.1016/j.ijpara.2022.04.002 PMC9250636

[5] Yu J, Yu Y, Li Q, Chen M, Shen H, Zhang R, Song M, Hu W (2019) Comprehensive analysis of miRNA profiles reveals the role of *Schistosoma japonicum* miRNAs at different developmental stages. Vet Res 50:23 10.1186/s13567-019-0642-2 30947738 10.1186/s13567-019-0642-2 PMC6449929

[6] McManus DP, Dunne DW, Sacko M, Utzinger J, Vennervald BJ, Zhou XN (2018) Schistosomiasis. Nat Rev Dis Primers 4:14. 10.1038/s41572-018-0013-8 30093684 10.1038/s41572-018-0013-8

[7] Chuah C, Jones MK, Burke ML, McManus DP, Gobert GN (2014) Cellular and chemokine-mediated regulation in schistosome-induced hepatic pathology. Trends Parasitol 30:141–150. 10.1016/j.pt.2013.12.009 24433721 10.1016/j.pt.2013.12.009

[8] Andrade ZA (2009) Schistosomiasis and liver fibrosis. Parasite Immunol 31:656–663. 10.1111/j.1365-3024.2009.01157.x 19825105 10.1111/j.1365-3024.2009.01157.x

[9] Carson JP, Ramm GA, Robinson MW, McManus DP, Gobert GN (2018) Schistosome-induced fibrotic disease: the role of hepatic stellate cells. Trends Parasitol 34:524–540. 10.1016/j.pt.2018.02.005 29526403 10.1016/j.pt.2018.02.005

[10] Mu Y, McManus DP, Gordon CA, Cai P (2021) Parasitic helminth-derived microRNAs and extracellular vesicle cargos as biomarkers for helminthic infections. Front Cell Infect Microbiol 11:708952. 10.3389/fcimb.2021.708952 34249784 10.3389/fcimb.2021.708952 PMC8267863

[11] van Niel G, D’Angelo G, Raposo G (2018) Shedding light on the cell biology of extracellular vesicles. Nat Rev Mol Cell Biol 19:213–228. 10.1038/nrm.2017.125 29339798 10.1038/nrm.2017.125

[12] Abou-El-Naga IF (2022) Emerging roles for extracellular vesicles in *Schistosoma* infection. Acta Trop 232:106467. 10.1016/j.actatropica.2022.106467 35427535 10.1016/j.actatropica.2022.106467

[13] Wang Y, Fan X, Lei N, He X, Wang X, Luo X, Zhang D, Pan W (2020) A MicroRNA derived from *Schistosoma japonicum* promotes schistosomiasis hepatic fibrosis by targeting host secreted frizzled-related protein 1. Front Cell Infect Microbiol 10:101. 10.3389/fcimb.2020.00101 32232014 10.3389/fcimb.2020.00101 PMC7082693

[14] Wang L, Liao Y, Yang R, Yu Z, Zhang L, Zhu Z, Wu X, Shen J, Liu J, Xu L, Wu Z, Sun X (2020) Sja-miR-71a in schistosome egg-derived extracellular vesicles suppresses liver fibrosis caused by schistosomiasis via targeting Semaphorin 4D. J Extracell Vesicles 9:1785738. 10.1080/20013078.2020.1785738 32944173 10.1080/20013078.2020.1785738 PMC7480424

[15] He X, Wang Y, Fan X, Lei N, Tian Y, Zhang D, Pan W (2020) A schistosome miRNA promotes host hepatic fibrosis by targeting transforming growth factor beta receptor III. J Hepatol 72:519–527. 10.1016/j.jhep.2019.10.029 31738999 10.1016/j.jhep.2019.10.029

[16] Chen Y, Hu Y, Zhou H, Jiang N, Wang Y, Zhang J, Shen Y, Yu G, Cao J (2024) Induction of hepatic fibrosis in mice with schistosomiasis by extracellular microRNA-30 derived from *Schistosoma japonicum* eggs. Front Immunol 15:1425384. 10.3389/fimmu.2024.1425384 39139565 10.3389/fimmu.2024.1425384 PMC11319242

[17] Wang Y, Gong W, Zhou H, Hu Y, Wang L, Shen Y, Yu G, Cao J (2022) A novel miRNA from egg-derived exosomes of *Schistosoma japonicum* promotes liver fibrosis in murine schistosomiasis. Front Immunol 13:860807. 10.3389/fimmu.2022.860807 35572578 10.3389/fimmu.2022.860807 PMC9094574

[18] Du P, Giri BR, Liu J, Xia T, Grevelding CG, Cheng G (2020). Proteomic and deep sequencing analysis of extracellular vesicles isolated from adult male and female *Schistosoma japonicum*. PLoS Negl Trop Dis 14:e0008618. 10.1371/journal.pntd.0008618. 32986706 10.1371/journal.pntd.0008618 PMC7521736

[19] Zhong H, Dong B, Zhu D, Fu Z, Liu J, Guan G, Jin Y (2025) RNA sequencing analysis reveals the impact of *Schistosoma japonicum* worm on host liver fibrosis. Acta Trop 270:107747. 10.1016/j.actatropica.2025.107747 40680920 10.1016/j.actatropica.2025.107747

[20] Smithers SR, Terry RJ (1965) The infection of laboratory hosts with cercariae of *Schistosoma mansoni* and the recovery of the adult worms. Parasitology 55:695–700. 10.1017/s0031182000086248 4957633 10.1017/s0031182000086248

[21] Zhong H, Dong B, Zhu D, Fu Z, Liu J, Jin Y (2024) Sja-let-7 suppresses the development of liver fibrosis via *Schistosoma japonicum* extracellular vesicles. PLoS Pathog 20:e1012153. 10.1371/journal.ppat.1012153 38598555 10.1371/journal.ppat.1012153 PMC11034668

[22] Zhu S, Wang S, Lin Y, Jiang P, Cui X, Wang X, Zhang Y, Pan W (2016) Release of extracellular vesicles containing small RNAs from the eggs of *Schistosoma japonicum*. Parasit Vectors 9:574. 10.1186/s13071-016-1845-2 27825390 10.1186/s13071-016-1845-2 PMC5101684

[23] Dong B, Zhong H, Zhu D, Li H, Lu K, Fu Z, Liu J, Jin Y (2025) Membrane proteomics and transcriptomic profiling analysis of hepatic stellate cells co-incubated with *Schistosoma japonicum* eggs. Front Cell Infect Microbiol 15:1674880. 10.3389/fcimb.2025.1674880 41036229 10.3389/fcimb.2025.1674880 PMC12479499

[24] White R, Sotillo J, Ancarola ME, Borup A, Boysen AT, Brindley PJ, Buzás EI, Cavallero S, Chaiyadet S, Chalmers IW, Cucher MA, Dagenais M, Davis CN, Devaney E, Duque‐Correa MA, Eichenberger RM, Fontenla S, Gasan TA, Hokke CH, Kosanovic M, Kuipers ME, Laha T, Loukas A, Maizels RM, Marcilla A, Mazanec H, Morphew RM, Neophytou K, Nguyen LT, Nolte‐‘t Hoen E, Povelones M, Robinson MW, Rojas A, Schabussova I, Smits HH, Sungpradit S, Tritten L, Whitehead B, Zakeri A, Nejsum P, Buck AH, Hoffmann KF (2023) Special considerations for studies of extracellular vesicles from parasitic helminths: a community‐led roadmap to increase rigour and reproducibility. J Extracell Vesicles 12:e12298. 10.1002/jev2.12298 36604533 10.1002/jev2.12298 PMC9816087

[25] Peterson SM, Thompson JA, Ufkin ML, Sathyanarayana P, Liaw L, Congdon CB (2014) Common features of microRNA target prediction tools. Front Genet 5:23. 10.3389/fgene.2014.00023 24600468 10.3389/fgene.2014.00023 PMC3927079

[26] Krüger J, Rehmsmeier M (2006) RNAhybrid: microRNA target prediction easy, fast and flexible. Nucleic Acids Res 34(Web Server issue):W451-454. 10.1093/nar/gkl243 16845047 10.1093/nar/gkl243 PMC1538877

[27] Conesa A, Götz S, García-Gómez JM, Terol J, Talón M, Robles M (2005) Blast2GO: a universal tool for annotation, visualization and analysis in functional genomics research. Bioinformatics 21:3674–3676. 10.1093/bioinformatics/bti610 16081474 10.1093/bioinformatics/bti610

[28] Zhou Y, Zhou B, Pache L, Chang M, Khodabakhshi AH, Tanaseichuk O, Benner C, Chanda SK (2019) Metascape provides a biologist-oriented resource for the analysis of systems-level datasets. Nat Commun 10:1523. 10.1038/s41467-019-09234-6 30944313 10.1038/s41467-019-09234-6 PMC6447622

[29] Li R, Li Y, Kristiansen K, Wang J (2008) SOAP: short oligonucleotide alignment program. Bioinformatics 24:713–714. 10.1093/bioinformatics/btn025 18227114 10.1093/bioinformatics/btn025

[30] Kim D, Langmead B, Salzberg SL (2015) HISAT: a fast spliced aligner with low memory requirements. Nat Methods 12:357–360. 10.1038/nmeth.3317 25751142 10.1038/nmeth.3317 PMC4655817

[31] Benelli M, Pescucci C, Marseglia G, Severgnini M, Torricelli F, Magi A (2012) Discovering chimeric transcripts in paired-end RNA-seq data by using EricScript. Bioinformatics 28:3232–3239. 10.1093/bioinformatics/bts617 23093608 10.1093/bioinformatics/bts617

[32] Shen S, Park JW, Lu ZX, Lin L, Henry MD, Wu YN, Zhou Q, Xing Y (2014) rMATS: robust and flexible detection of differential alternative splicing from replicate RNA-seq data. Proc Natl Acad Sci U S A 111:E5593–E5601. 10.1073/pnas.1419161111 25480548 10.1073/pnas.1419161111 PMC4280593

[33] Langmead B, Salzberg SL (2012) Fast gapped-read alignment with Bowtie 2. Nat Methods 9:357–359. 10.1038/nmeth.1923 22388286 10.1038/nmeth.1923 PMC3322381

[34] Love MI, Huber W, Anders S (2014) Moderated estimation of fold change and dispersion for RNA-seq data with DESeq2. Genome Biol 15:550. 10.1186/s13059-014-0550-8 25516281 10.1186/s13059-014-0550-8 PMC4302049

[35] Reinholdt C, Winkelmann F, Koslowski N, Reisinger EC, Sombetzki M (2023) Unisexual infection with *Schistosoma mansoni* in mice has the potential to boost the immune response against eggs after challenge infection. Front Immunol 14:1125912. 10.3389/fimmu.2023.1125912 36923416 10.3389/fimmu.2023.1125912 PMC10009330

[36] Livak KJ, Schmittgen TD (2001) Analysis of relative gene expression data using real-time quantitative PCR and the 2(-delta delta C(T)) method. Methods 25:402–408. 10.1006/meth.2001.1262 11846609 10.1006/meth.2001.1262

[37] Weiskirchen R, Tacke F (2014) Cellular and molecular functions of hepatic stellate cells in inflammatory responses and liver immunology: Hepatobiliary Surg Nutr 3:344–363. 10.3978/j.issn.2304-3881.2014.11.03 25568859 10.3978/j.issn.2304-3881.2014.11.03 PMC4273119

[38] Liu M, Cho WC, Flynn RJ, Jin X, Song H, Zheng Y (2023) microRNAs in parasite-induced liver fibrosis: from mechanisms to diagnostics and therapeutics. Trends Parasitol 39:859–872. 10.1016/j.pt.2023.07.001 37516634 10.1016/j.pt.2023.07.001

[39] Elsharkawy AM, Oakley F, Mann DA (2005) The role and regulation of hepatic stellate cell apoptosis in reversal of liver fibrosis. Apoptosis 10:927–939. 10.1007/s10495-005-1055-4 16151628 10.1007/s10495-005-1055-4

[40] Lee SW, Kim SM, Hur W, Kang BY, Lee HL, Nam H, Yoo SH, Sung PS, Kwon JH, Jang JW, Kim SJ, Yoon SK (2021) Tenofovir disoproxil fumarate directly ameliorates liver fibrosis by inducing hepatic stellate cell apoptosis via downregulation of PI3K/Akt/mTOR signaling pathway. PLoS One 16:e0261067. 10.1371/journal.pone.0261067 34879114 10.1371/journal.pone.0261067 PMC8654182

[41] Tan Z, Sun H, Xue T, Gan C, Liu H, Xie Y, Yao Y, Ye T (2021) Liver fibrosis: therapeutic targets and advances in drug therapy. Front Cell Dev Biol 9:730176. 10.3389/fcell.2021.730176 34621747 10.3389/fcell.2021.730176 PMC8490799

[42] Meng D, Li Z, Wang G, Ling L, Wu Y, Zhang C (2018) Carvedilol attenuates liver fibrosis by suppressing autophagy and promoting apoptosis in hepatic stellate cells. Biomed Pharmacother 108:1617–1627. 10.1016/j.biopha.2018.10.005 30372864 10.1016/j.biopha.2018.10.005

[43] Han H, Peng J, Hong Y, Fu Z, Lu K, Li H, Zhu C, Zhao Q, Lin J (2015) Comparative characterization of microRNAs in *Schistosoma japonicum* schistosomula from Wistar rats and BALB/c mice. Parasitol Res 114:2639–2647. 10.1007/s00436-015-4468-1 25895062 10.1007/s00436-015-4468-1

[44] Lee EF, Young ND, Lim NTY, Gasser RB, Fairlie WD (2014) Apoptosis in schistosomes: toward novel targets for the treatment of schistosomiasis. Trends Parasitol 30:75–84. 10.1016/j.pt.2013.12.005 24393571 10.1016/j.pt.2013.12.005

[45] Wang T, Guo X, Hong Y, Han H, Cao X, Han Y, Zhang M, Wu M, Fu Z, Lu K, Li H, Zhao Z, Lin J (2016) Comparison of apoptosis between adult worms of *Schistosoma japonicum* from susceptible (BALB/c mice) and less-susceptible (Wistar rats) hosts. Gene 592:71–77. 10.1016/j.gene.2016.07.054 27461946 10.1016/j.gene.2016.07.054

[46] Marco A, Kozomara A, Hui JH, Emery AM, Rollinson D, Griffiths-Jones S, Ronshaugen M (2013) Sex-biased expression of microRNAs in *Schistosoma mansoni*. PLoS Negl Trop Dis 7:e2402. 10.1371/journal.pntd.0002402 24069470 10.1371/journal.pntd.0002402 PMC3772069

[47] Hoy AM, Lundie RJ, Ivens A, Quintana JF, Nausch N, Forster T, Jones F, Kabatereine NB, Dunne DW, Mutapi F, Macdonald AS, Buck AH (2014) Parasite-derived microRNAs in host serum as novel biomarkers of helminth infection. PLoS Negl Trop Dis 8:e2701. 10.1371/journal.pntd.0002701 24587461 10.1371/journal.pntd.0002701 PMC3930507

[48] Stroehlein AJ, Young ND, Korhonen PK, Hall RS, Jex AR, Webster BL, Rollinson D, Brindley PJ, Gasser RB (2018) The small RNA complement of adult *Schistosoma haematobium*. PLoS Negl Trop Dis 12:e0006535. 10.1371/journal.pntd.0006535 29813122 10.1371/journal.pntd.0006535 PMC5993326

[49] Mu Y, Cai P, Olveda RM, Ross AG, Olveda DU, McManus DP (2019) Parasite-derived circulating microRNAs as biomarkers for the detection of human *Schistosoma japonicum* infection. Parasitology 147:889–896. 10.1017/S0031182019001690 31840631 10.1017/S0031182019001690 PMC7391863

[50] Ovchinnikov VY, Kashina EV, Mordvinov VA, Fromm B (2020) EV-transported microRNAs of *Schistosoma mansoni* and *Fasciola hepatica*: potential targets in definitive hosts. Infect Genet Evol 85:104528. 10.1016/j.meegid.2020.104528 32891875 10.1016/j.meegid.2020.104528

[51] Diaz A, Macchiaroli N, Preza M, Pérez MG, Kamenetzky L, Cucher M, Koziol U, Castillo E, Berriman M, Brehm K, Rosenzvit MC (2021) Expression profiling of *Echinococcus multilocularis* miRNAs throughout metacestode development in vitro. PLoS Negl Trop Dis 15:e0009297. 10.1371/journal.pntd.0009297 33750964 10.1371/journal.pntd.0009297 PMC8016320

[52] Karami MF, Beiromvand M, Rafiei A, Dayer D, Rahdar M, Bahreini A, Dastyar AA (2022) Serum level of egr-miR-2a-3p as a potential diagnostic biomarker for cystic echinococcosis. Acta Parasitol 68(1):114–121. 10.1007/s11686-022-00641-2 36434379 10.1007/s11686-022-00641-2

[53] Niu X, Chang G, Xu N, Li R, Niu B, Mao R, Wang S, Li G, Jiang J, Wang L (2025) Vitamin A-integrated cinnamaldehyde nanoemulsion: a nanotherapeutic approach to counteract liver fibrosis via gut-liver axis modulation. ACS Nano 9:10433–10451. 10.1021/acsnano.5c00136 10.1021/acsnano.5c00136 40045827

[54] Rettkowski J, Romero-Mulero MC, Singh I, Wadle C, Wrobel J, Chiang D, Hoppe N, Mess J, Schönberger K, Lalioti ME, Jäcklein K, SilvaRego B, Bühler T, Karabacz N, Egg M, Demollin H, Obier N, Zhang YW, Jülicher C, Hetkamp A, Czerny M, Jones MJ, Seung H, Jain R, von Zur Mühlen C, Maier A, Lother A, Hilgendorf I, van Galen P, Kreso A, Westermann D, Rodriguez-Fraticelli AE, Heidt T, Cabezas-Wallscheid N (2025) Modulation of bone marrow haematopoietic stem cell activity as a therapeutic strategy after myocardial infarction: a preclinical study. Nat Cell Biol 27:591–604. 10.1038/s41556-025-01639-4 40175666 10.1038/s41556-025-01639-4 PMC11991920

[55] Zhong H, Dong B, Zhu D, Li H, Lu K, Fu Z, Liu J, Jin Y (2023) Sja-Let-7 attenuates carbon tetrachloride-induced liver fibrosis in a mouse model via Col1α2. Biology 12:1465. 10.3390/biology12121465 38132291 10.3390/biology12121465 PMC10740823

