# Supplementary material for: Host–parasite cross-species regulation by Schistosoma japonicum EV-miRNA sja-miR-2a-3p limits hepatic fibrosis via BCL2 suppression in hepatic stellate cells
Source: Vet Res. 2026 Aug 14;57:150. doi: 10.1186/s13567-026-01794-y (PMC13476918; doi:10.1186/s13567-026-01794-y)
Supplement: Supplementary file 1 [file 13567_2026_1794_MOESM18_ESM.pdf]

Original figure of western blot analysis

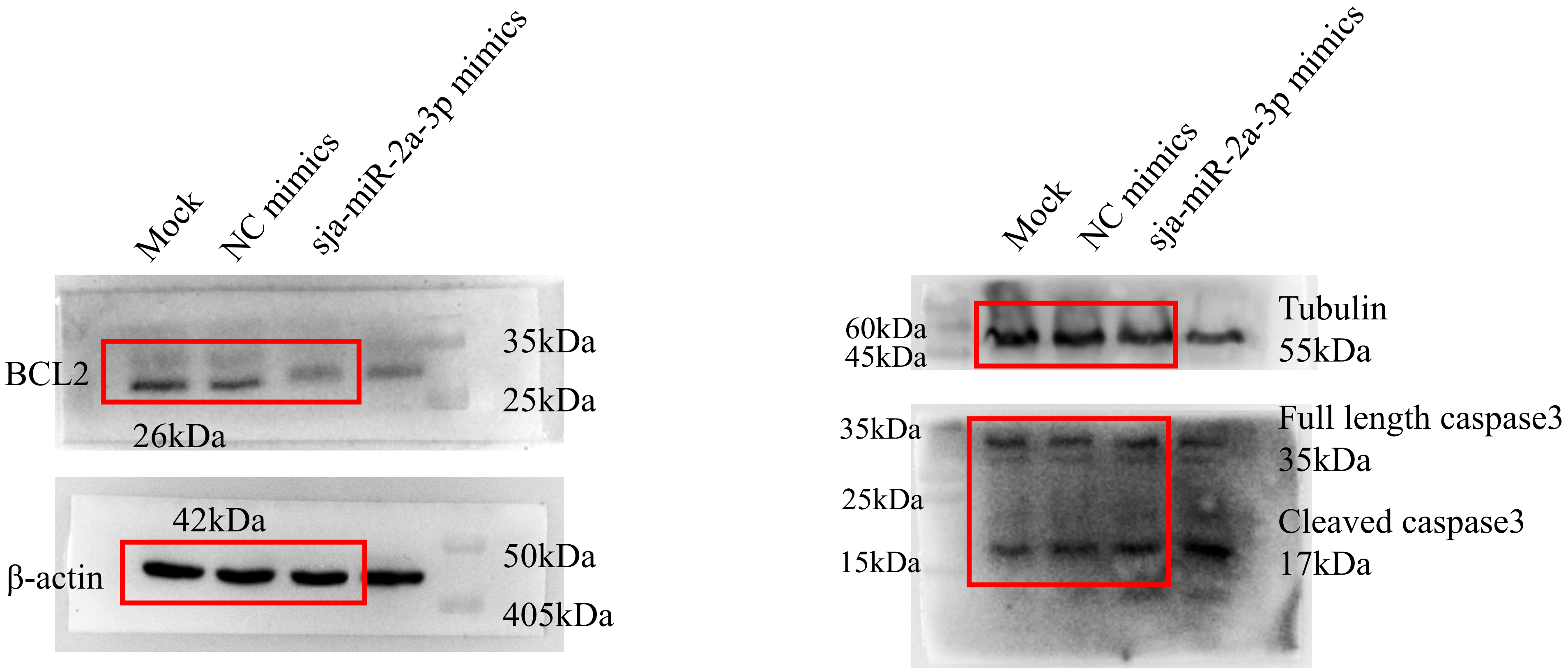

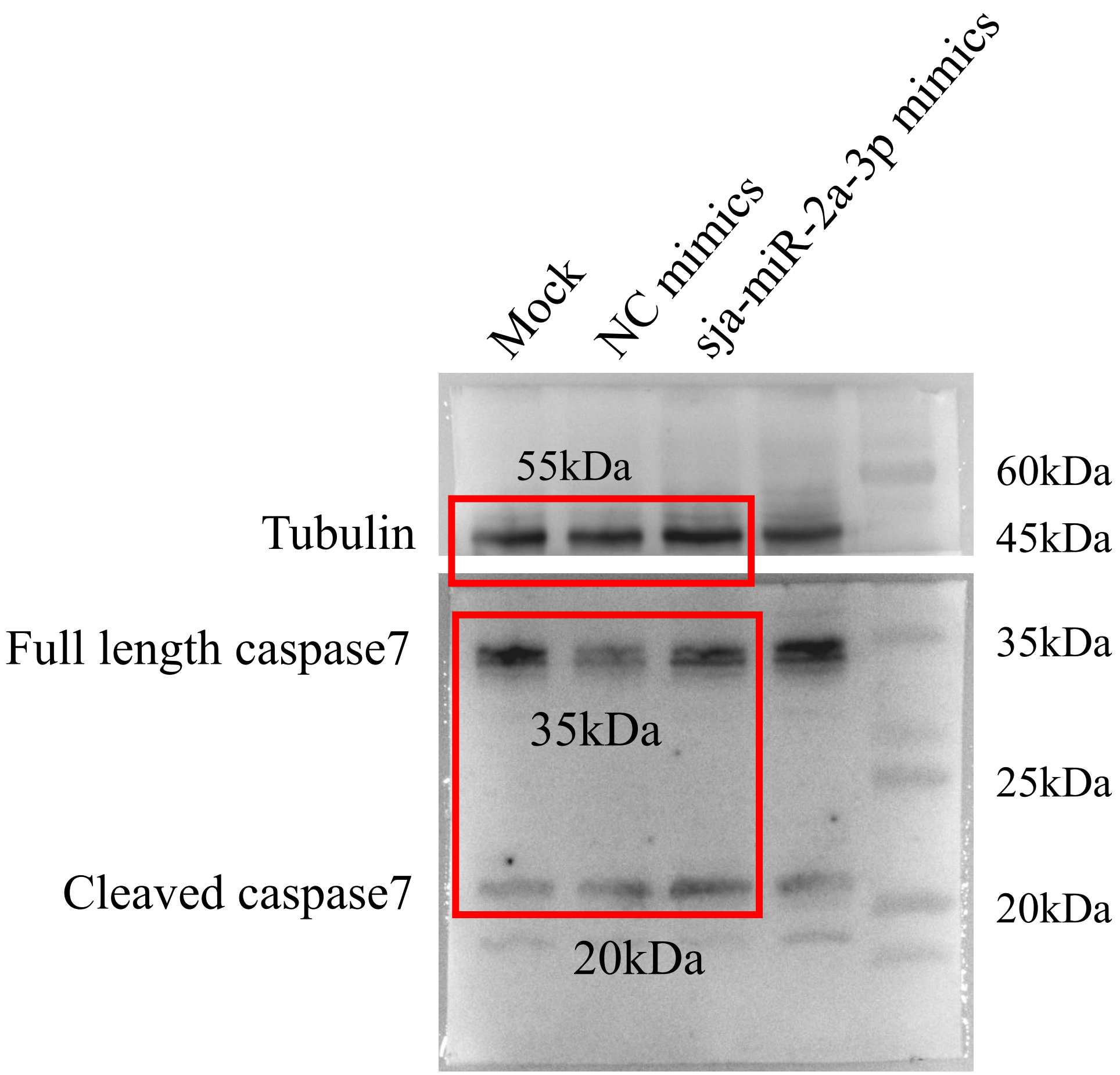
Mock
NC mimics
sja-miR-2a-3p mimics
55kDa
60kDa
Tubulin
45kDa
Full length caspase7
35kDa
35kDa
25kDa
Cleaved caspase7
20kDa
20kDa

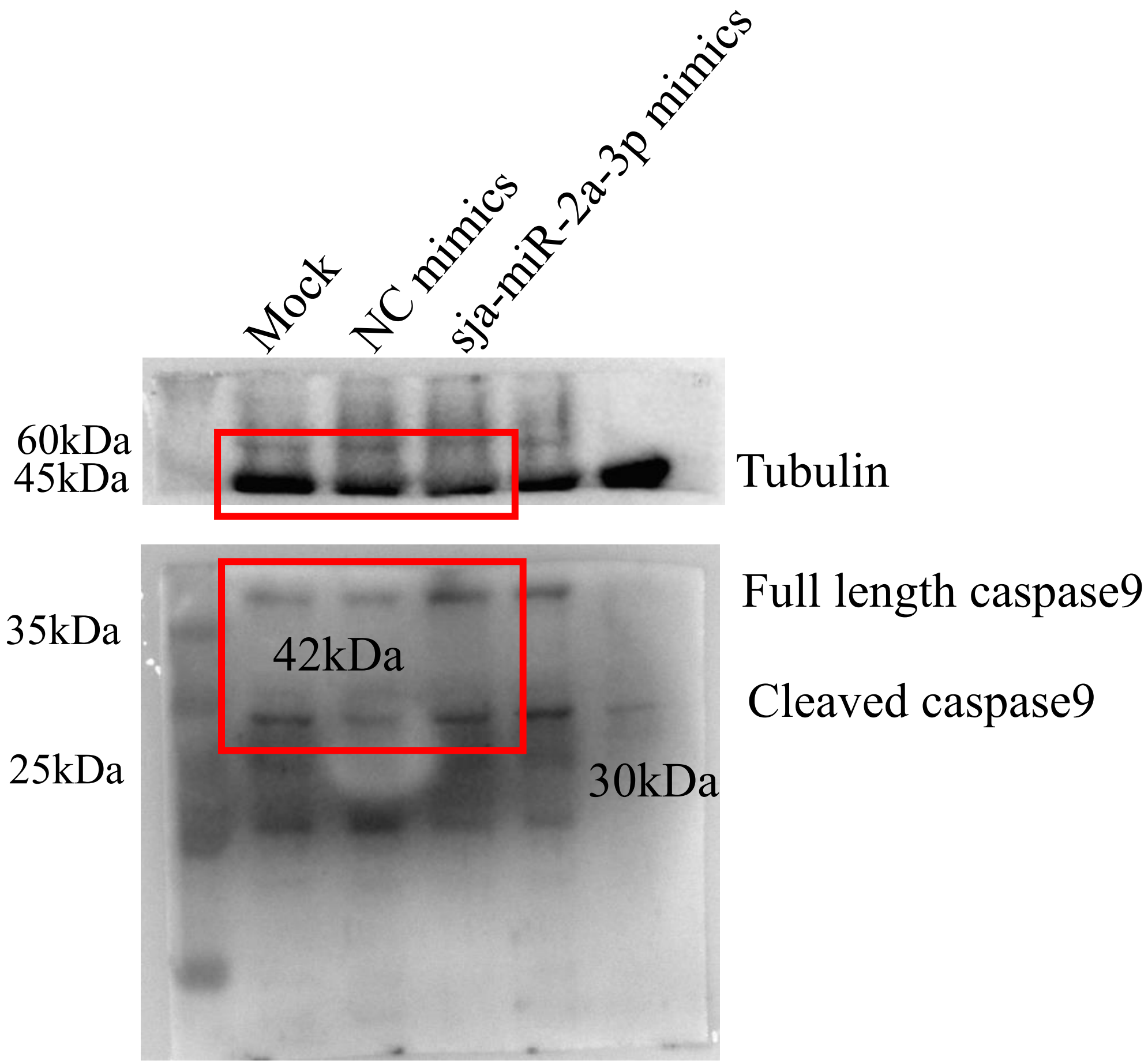
Mock
NC mimics
sja-miR-2a-3p mimics
60kDa
45kDa
Tubulin
Full length caspase9
35kDa
42kDa
Cleaved caspase9
25kDa
30kDa
