# Supplementary material for: Host–parasite cross-species regulation by Schistosoma japonicum EV-miRNA sja-miR-2a-3p limits hepatic fibrosis via BCL2 suppression in hepatic stellate cells
Source: Vet Res. 2026 Aug 14;57:150. doi: 10.1186/s13567-026-01794-y (PMC13476918; doi:10.1186/s13567-026-01794-y)
Supplement: Supplementary file 2 [file 13567_2026_1794_MOESM1_ESM.docx]

Additional file 1. The sequence of miRNA mimics, inhibitor and agomir.

| Name | Sense sequence (5'-3') | Anti-sense sequence (5'-3') |
| --- | --- | --- |
| sja-miR-2a-3p inhibitor | CGUUCAUCAAUACUGGCUGUGA | / |
| NC inhibitor | CAGUACUUUUGUGUAGUACAA | / |
| sja-miR-2a-3p agomir | UCACAGCCAGUAUUGAUGAACG | UUCAUCAAUACUGGCUGUGAUU |
| NC agomir | UUCUCCGAACGUGUCACGUTT | ACGUGACACGUUCGGAGAATT |
| sja-miR-2a-3p mimics | UCACAGCCAGUAUUGAUGAACG | UUCAUCAAUACUGGCUGUGAUU |
| NC mimics | UUCUCCGAACGUGUCACGUTT | ACGUGACACGUUCGGAGAATT |
