# Supplementary material for: Host–parasite cross-species regulation by Schistosoma japonicum EV-miRNA sja-miR-2a-3p limits hepatic fibrosis via BCL2 suppression in hepatic stellate cells
Source: Vet Res. 2026 Aug 14;57:150. doi: 10.1186/s13567-026-01794-y (PMC13476918; doi:10.1186/s13567-026-01794-y)
Supplement: Supplementary file 3 [file 13567_2026_1794_MOESM2_ESM.docx]

**Additional files 2. Details for *Sj*EVs collection and function analysis**

| Titles | Details |
| --- | --- |
| Helminths | |
| Helminth species | *Schistosoma japonicum* Anhui strain |
| Lifestage | 30 days |
| Number of worms | ~15 worm pairs per mL of media |
| Washing buffer | Phosphate buffer solution (PBS, Corning, USA) |
| Host |  |
| Host species | Balb/c mouse |
| Host age and sex | 7–8 weeks old; male |
| Host tissue from where the worms were isolated | Hepatic portal vein |
| Helminth cultivation |  |
| Culture length | 2 hours |
| Composition of culture media | RPMI-1640 (Corning, USA) containing 1% penicillin-streptomycin (Thermo Fisher Scientific, USA) |
| Number of worms per mL of media | 15 worm pairs |
| Incubation temperature and % CO2 | 37°C, 5% CO_2_ |
| Parasite viability | Visual inspection |
| Number of parasite pre-incubation washes | 3–5 times with 50 mL PBS |
| EV separation and QC |  |
| ES collection timepoints | Every 2 hours during cultivation |
| EV storage details | -80 °C |
| EV separation technique used and details | After 2 h incubation, the supernatant was collected and fresh culture medium was added for the next collection (the whole collecting procedure could last for 3–4 days until the worms were less active). The pellets in the collected supernatant were discarded by centrifugation at 3,000× g at 4°C, followed by filtration through a 0.22 μm syringe filter (Pall, USA). The filtrates were then concentrated to a final volume of 1 mL using the Exosupur® High Concentration Kit for Cell Culture Supernatants (Echo Biotech, China). Purification of *Sj*EVs was subsequently performed using the Exosupur® Exosome Purification Kit (Echo Biotech, China). The first three fractions, which did not contain EVs, were designated as *Sj*EV-depleted excretory/secretory products (ESPs). The following five fractions (fractions 4–8, total volume: 2.5 mL) were pooled as *Sj*EV-containing fractions. These *Sj*EV fractions were further concentrated to approximately 200 μL using a 100 kDa molecular weight cutoff centrifugal filter (Sigma, USA) unit at 4,000 × g. Final samples were stored at –80 ℃ until further use. |
| Details of low speed differential centrifugation to pre-clear ES of eggs and debris | To pre-clear ES of eggs and debris. The pellets in the collected supernatant were discarded by centrifugation at 3,000× g for 10 min each at 4°C. |
| Functional studies of helminth EVS |  |
| Measurement of number of particles | 1.64×10^11^/ mL |
| Size distribution | 83.6±24.7 nm |
| Controls used for functional studies and how they were obtained | *Sj*EV-depleted ESPs (Excretory-secretory products), the supernatant after pelleting the *Sj*EVs was collected |
| Normalisation of EVs for functional studies (number of vesicles/mL) | 2.96×10^9^ particles/mL |
