# Supplementary material for: Host–parasite cross-species regulation by Schistosoma japonicum EV-miRNA sja-miR-2a-3p limits hepatic fibrosis via BCL2 suppression in hepatic stellate cells
Source: Vet Res. 2026 Aug 14;57:150. doi: 10.1186/s13567-026-01794-y (PMC13476918; doi:10.1186/s13567-026-01794-y)
Supplement: Supplementary file 4 [file 13567_2026_1794_MOESM3_ESM.docx]

Additional file 3. Antibodies used in the experiment

| Experiments | Primary antibody | Source  (Catalogue No.) | Host | Working conditions | Secondary antibody | Source (Catalogue No.) | Working conditions |
| --- | --- | --- | --- | --- | --- | --- | --- |
| Western blot assay | BCL2 | Bioss  (bs-0032R) | Rabbit | 1:1500 | HRP conjugated Goat Anti-Rabbit IgG (H+L) | Cell signaling  (7074S) | 1:3000 |
|  | β-actin | Servicebio  (GB15001) | Rabbit | 1:3000 |  |  |  |
|  | Caspase 7 | Proteintech  (27155-1-AP) | Rabbit | 1:800 |  |  |  |
|  | Caspase 9 | Proteintech  (10380-1-AP) | Rabbit | 1:500 |  |  |  |
|  | β-Tubulin | Servicebio  (GB15140) | Mouse | 1:3000 | HRP conjugated Goat Anti-Mouse IgG (H+L) | Cell signaling  (7076S) | 1:3000 |
|  | Caspase 3 | Proteintech  (66470-2-Ig) | Mouse | 1:2000 |  |  |  |
| Immunofluorescence analysis | Desmin | Servicebio  (GB12081) | Mouse | 1:200 | Cy3 conjugated Goat Anti-Rabbit IgG (H+L) | Servicebio  (GB21301) | 1:300 |
|  | Bad | Servicebio  (GB11198) | Rabbit | 1:300 | Alexa Fluor® 488-conjugated Goat Anti-Rabbit IgG (H+L) | Servicebio  (GB25303) | 1:400 |
|  | FasL | Servicebio  (GB11090-1) | Rabbit | 1:500 |  |  |  |
| Immunohistochemical assay | α-SMA | Servicebio  (GB111364) | Rabbit | 1:2000 | HRP conjugated Goat Anti-Rabbit IgG (H+L) | Servicebio  (GB23303) | 1:200 |
|  | Col1α1 | Servicebio  (GB11022) | Rabbit | 1:1000 |  |  |  |
|  | Col3α1 | Servicebio  (GB111629) | Rabbit | 1:500 |  |  |  |
