# Supplementary material for: Host–parasite cross-species regulation by Schistosoma japonicum EV-miRNA sja-miR-2a-3p limits hepatic fibrosis via BCL2 suppression in hepatic stellate cells
Source: Vet Res. 2026 Aug 14;57:150. doi: 10.1186/s13567-026-01794-y (PMC13476918; doi:10.1186/s13567-026-01794-y)
Supplement: Supplementary file 5 [file 13567_2026_1794_MOESM4_ESM.docx]

Additional files 4. Probes used in the FISH analysis.

| Probe name | Sequence |
| --- | --- |
| sja-miR-2a-3p | 5’-CGTTCATCAATACTGGCTGTGA-3’ |
| BCL2 | 5’-CACGATCTCCCGGTTATCATACCC-3’ |
|  | 5’-TGAGAGGAGACGTCCTGGCAGCCA-3’ |
|  | 5’-CAGGTGCAGCTGACTGGACATCTC-3’ |
|  | 5’-TGCAGATGCCGGTTCAGGTACTCA-3’ |
