# Supplementary material for: Host–parasite cross-species regulation by Schistosoma japonicum EV-miRNA sja-miR-2a-3p limits hepatic fibrosis via BCL2 suppression in hepatic stellate cells
Source: Vet Res. 2026 Aug 14;57:150. doi: 10.1186/s13567-026-01794-y (PMC13476918; doi:10.1186/s13567-026-01794-y)
Supplement: Supplementary file 6 [file 13567_2026_1794_MOESM5_ESM.docx]

Additional files 5. Primers used in the experiment

| Primer | Sequences (5’-3’) |
| --- | --- |
| Human β-actin-F | GATGTGGATCAGCAAGCAGGAG |
| Human β-actin-R | AGAAAGGGTGTAACGCAACTAAGTC |
| Human Bcl-2-F | TTGTTGTTGTTCAAACGGGATTCAC |
| Human Bcl-2-R | GCAGCCAGCCAGCAATTAGC |
| Mouse-IL-1β-F | ATGAAAGACGGCACACCCAC |
| Mouse-IL-1β-R | GCTTGTGCTCTGCTTGTGAG |
| Mouse-IL-6-F | TGCAAGAGACTTCCATCCAGT |
| Mouse-IL-6-R | GTGAAGTAGGGAAGGCCG |
| Mouse-TNFα-F | CAGCCGATGGGTTGTACCTT |
| Mouse-TNFα-R | TGTGGGTGAGGAGCACGTAGT |
| Mouse-TLR4-F | TAGCACAGAAGAGGCAAGGC |
| Mouse-TLR4-R | GACCCTGACTGGCACTAACC |
| Mouse-α-SMA-F | TCAGCGCCTCCAGTTCCT |
| Mouse-α-SMA-R | AAAAAAAACCACGAGTAACAAATCAA |
| Mouse-Col1α1-F | ACGTCCTGGTGAAGTTGGTC |
| Mouse-Col1α1-R | CAGGGAAGCCTCTTTCTCCT |
| Mouse-Col3α1-F | GCCCACAGCCTTCTACACCT |
| Mouse-Col3α1-R | GCCAGGGTCACCATTTCTC |
| Mouse-TGF-β1-F | ATTCCTGGCGTTACCTTGG |
| Mouse-TGF-β1-R | AGCCCTGTATTCCGTCTCCT |
| Mouse-Smad3-F | GAGACATTCCACGCTTCACA |
| Mouse-Smad3-R | GCTGCATTCCGGTTAACATT |
| sja-miR-2a-3p-RT-primer | GTCGTATCCAGTGCAGGGTCCGAGGTATTCGCACTGGATACGACCGTTCA |
| sja-miR-2a-3p-Forward | AAGCGCCTTCACAGCCAGTATT |
| sja-miR-2a-3p-Reverse | ATCCAGTGCAGGGTCCGAGG |
| U6-RT-primer | GTCGTATCCAGTGCAGGGTCCGAGGTATTCGCACTGGATACGACAAAATATG |
| U6-Forward | CTCGCTTCGGCAGCACA |
| U6-Reverse | AACGCTTCACGAATTTGCGT |
